# Enhancing CRISPR-Cas-based gene targeting in tomato using a dominant-negative *ku80*

**DOI:** 10.1093/hr/uhae294

**Published:** 2024-10-23

**Authors:** Tien Van Vu, Ngan Thi Nguyen, Jihae Kim, Minh Huy Vu, Young Jong Song, Mil Thi Tran, Yeon Woo Sung, Jae-Yean Kim

**Affiliations:** Division of Applied Life Science (BK21 Four Program), Plant Molecular Biology and Biotechnology Research Center, Gyeongsang National University, Jinju 660-701, Republic of Korea; Division of Applied Life Science (BK21 Four Program), Plant Molecular Biology and Biotechnology Research Center, Gyeongsang National University, Jinju 660-701, Republic of Korea; Division of Applied Life Science (BK21 Four Program), Plant Molecular Biology and Biotechnology Research Center, Gyeongsang National University, Jinju 660-701, Republic of Korea; Division of Applied Life Science (BK21 Four Program), Plant Molecular Biology and Biotechnology Research Center, Gyeongsang National University, Jinju 660-701, Republic of Korea; Division of Applied Life Science (BK21 Four Program), Plant Molecular Biology and Biotechnology Research Center, Gyeongsang National University, Jinju 660-701, Republic of Korea; Division of Applied Life Science (BK21 Four Program), Plant Molecular Biology and Biotechnology Research Center, Gyeongsang National University, Jinju 660-701, Republic of Korea; Current affiliation: Biological Resource Center, Korea Research Institute of Bioscience and Biotechnology (KRIBB), Jeongeup 56212, Republic of Korea; Division of Applied Life Science (BK21 Four Program), Plant Molecular Biology and Biotechnology Research Center, Gyeongsang National University, Jinju 660-701, Republic of Korea; Division of Applied Life Science (BK21 Four Program), Plant Molecular Biology and Biotechnology Research Center, Gyeongsang National University, Jinju 660-701, Republic of Korea; Division of Life Science, Gyeongsang National University, 501 Jinju-daero, Jinju 52828, Republic of Korea; Nulla Bio Inc 501 Jinju-daero, Jinju 52828, Republic of Korea

## Abstract

The CRISPR-Cas-based gene targeting (GT) method has enabled precise modifications of genomic DNA ranging from single base to several kilobase scales through homologous recombination (HR). In plant somatic cells, canonical non-homologous end-joining (cNHEJ) is the predominant mechanism for repairing double-stranded breaks (DSBs), thus limiting the HR-mediated GT. In this study, we implemented an approach to shift the repair pathway preference toward HR by using a dominant-negative *ku80* mutant protein (KUDN) to disrupt the initiation of cNHEJ. The employment of KUDN conferred a 1.71- to 3.55-fold improvement in GT efficiency at the callus stage. When we screened transformants, there was a more remarkable increase in GT efficiency, ranging from 1.62- to 9.84-fold, at two specific tomato loci, *SlHKT1;2* and *SlEPSPS1*. With practical levels of efficiency, this enhanced KUDN-based GT tool successfully facilitated a 9-bp addition at an additional locus, *SlCAB13*. These findings provide another promising method for more efficient and precise plant breeding.

## Introduction

Gene targeting (GT) was initially designed to replace a genomic DNA sequence with exogenous DNA donors through the homologous recombination (HR) mechanism. GT is one of the few methods capable of precisely editing genes of interest across scales ranging from single base pairs to kilobases while avoiding any undesirable alterations to the genome [1, 2]. While the prime editing technique shows promise for similar precision, it remains confined to small-scale DNA modifications without any scar [3–5]. GT holds the potential to meticulously replace entire genes or alleles or enable complex edit installation, offering a valuable strategy for gene/allele pyramiding in precision plant breeding [1]. In plants, the GT efficiency has been notably low for practical applications [2, 6].

Two major strategies that could enhance the GT efficiency in plants are (1) leveraging artificial DNA double-stranded breaks (DSBs) and (2) introducing a substantial quantity of DNA donors [7]. The efficiency of HR has been boosted from 10 to 100 times by employing the meganuclease I-Sce I to induce site-specific DSBs. Recently, various approaches have been presented to improve GT rates. Firstly, molecular scissors such as the Clustered Regularly Interspaced Short Palindromic Repeats (CRISPR)-CRISPR-associated protein (Cas) are utilized to induce DSBs at the intended target sequences [6–9]. Secondly, the frequency of HR is heightened by incorporating a viral replicon vehicle for donor templates [6, 7, 10, 11]. Likewise, several novel approaches have been reported in supporting elevated GT efficiency in mammals and plants, including the facilitating the HR pathway choice via suppression of the canonical nonhomologous end-joining (cNHEJ) by chemical [12, 13] or biological agents [14–18] and facilitation of DSB end resection by introducing DSB end processing enzymes [19, 20].

Previously, we established a GT system based on a geminiviral replicon and the CRISPR-LbCas12a nuclease [6] that was improved by using the temperature-tolerant (ttLbCas12a) variant [13, 21–23] and further enhanced by chemical treatments for cNHEJ suppression [13]. However, it is still challenging to conduct GT experiments without allele-associated markers for selecting GT events, which limits the applications of GT in tomatoes and other plants. We hypothesize that our GT system can be further improved by updating with the recent advancements in favoring the HR choice for DSB repair using a defective variant of the ku80 protein, a major player in the cNHEJ processing. Our data indicates that the GT efficiency was enhanced by 1.71- to 3.55-fold and 1.62- to 9.84-fold at the callus and plant stages, respectively, using the KUDN-based GT tool. Subsequently, the tool was applicable to other loci at practical levels, though its efficiency is still genomic context-dependent.

## Results

### NKUDN enhanced GT efficiency

**Figure 1:**
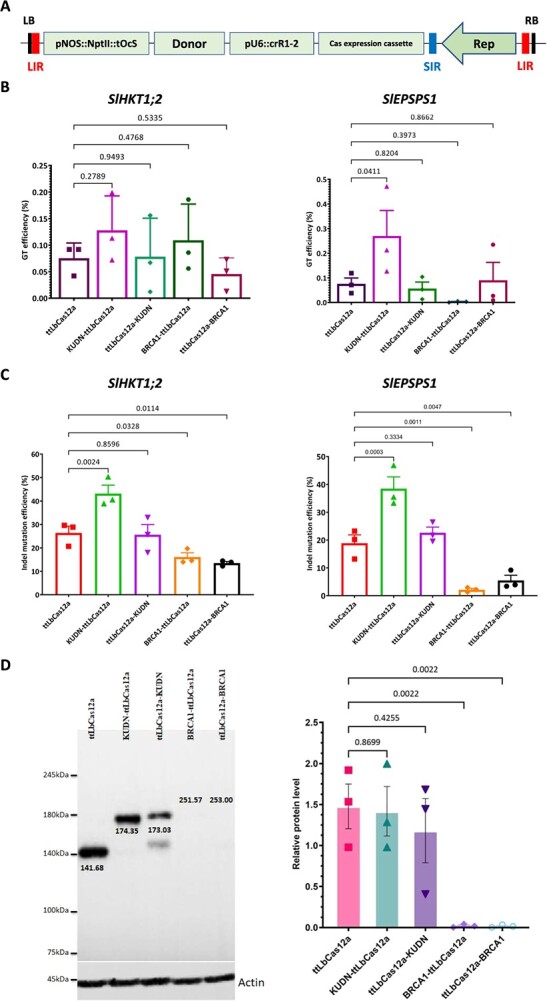
Impacts of the factors that affect DSB repair pathway choice on the editing efficiency in tomato. **A,** Plasmid map of the GT tool used in the study. The GT construct was cloned into a geminiviral replicon vector with the boundaries of two long intergenic sequences (LIRs) and one short intergenic sequence (SIR). The replicon is autonomously replicated with the support of the Rep protein/expression cassette. **B–C,** The impacts of KUDN and BRCA1 on GT (**B**) and indel mutation (**C**) efficiency at the callus stage. The efficiencies were assessed by targeted NGS using 21-dpt cotyledon/callus samples. **D,** The protein expression levels of ttLbCas12a and its KUDN and BRCA1 fusions. Western blot membranes showing bands of the Cas proteins with expected sizes and expression cassettes. Actin levels were used as loading controls. *P*-values of the *t*-test for pairwise comparison between the relative protein levels of ttLbCas12a and the fusions are indicated on the top of the bars. All data points are shown as dots in the plots in (**B–D**). The expression cassettes and Cas configurations are at the bottom of the bars.

The Ku70/80 complex was shown to play roles in early-sensing DSBs, binding and activating the cNHEJ pathway [24]. Knocking out or blocking the Ku70/80 complex with chemicals led to the enhancement of the alternative pathways, including the HR [12, 14, 15, 25, 26]. A truncated *ku80* (449–732) fragment showed a dominant-negative activity that interferes with the NHEJ pathway in hamster [27]. We identified the tomato Ku80 homolog (Solyc01g091350) and a putative *SlKu80DN* (427–709) fragment (named KUDN) by NCBI BLAST (Fig. S1). We hypothesized that this KUDN could increase the plant's HR-based GT. By fusing KUDN to ttLbCas12a at the N-terminal (termed NKUDN) and C-terminal (CKUDN), the KUDN peptide is directly located at the targeted sites for effective actions. We designed and tested GT tools (Fig. 1a) using the KUDN by fusing them to the ttLbCas12a protein (Fig. S2) for carrying them to the target sites (*SlHKT1;2* and *SlEPSPS1*). The targeted deep-sequencing data obtained from analyzing 10-day post-transformation (dpt) explants were initially used to reveal the impacts of the fusions on editing efficiencies.

We observed the improvement of HR-based GT efficiency at the callus stage, which was revealed by targeted NGS using NKUDN but not CKUDN configuration (Fig. 1b). More importantly, NKUDN significantly enhanced the GT efficiency at the *SlEPSPS1* locus compared to the construct without KUDN (0.270 ± 0.103% vs 0.076 ± 0.024%, a 3.55-fold increase). NKUDN also helped to improve GT at the *SlHKT1;2* site from 0.075 ± 0.017% to 0.128 ± 0.037%, a 1.71-fold enhancement (Fig. 1b). The GT efficiency reached up to 0.199% and 0.471% at the *SlHKT1;2*, and *SlEPSPS1* sites, respectively, with the involvement of the KUDN-ttLbCas12a module (Fig. 1b). This data indicates that NKUDN positively impacted GT, albeit the GT efficiency differed among targeted sites. Subsequently, we found that NKUDN caused higher indel mutation efficiency to both the *SlHKT1;2* and *SlEPSPS1* targets (Fig. 1c).

We reasoned that the fusions of KUDN to ttLbCas12a could alter the protein's expression levels, thereby affecting the GT efficiency. Using western blot analysis, we found that the relative protein levels of the fusions were not significantly changed between the ttLbCas12a (1.478 ± 0.273) and KUDN-ttLbCas12a (1.418 ± 0.301) (Fig. 1d). This indicates that the enhanced GT and indel mutation of the NKUDN could be attributed to the KUDN activity.

### The KUDN involvement led to a notable increase in MMEJ repair

A DSB in DNA can be repaired through two main pathways: cNHEJ and HDR. Among the sub-pathways of HDR, MMEJ, also known as alternative NHEJ, is an error-prone repair mechanism that requires short microhomologies (MHs) flanking the DSB ends [28]. These sequences anneal to seal the break, causing a deletion mutation that loses an MH and the sequence between the MHs [29]. The MMEJ repair mechanism shares a common initiation step with HR, which requires the redirection of the DSB repair pathway choice from cNHEJ to HDR by initiating DSB end resection [13, 28, 30]. To determine if the addition of KUDN could impair cNHEJ and enhance MMEJ, we analyzed the MMEJ traces from the targeted NGS data.

**Figure 2:**
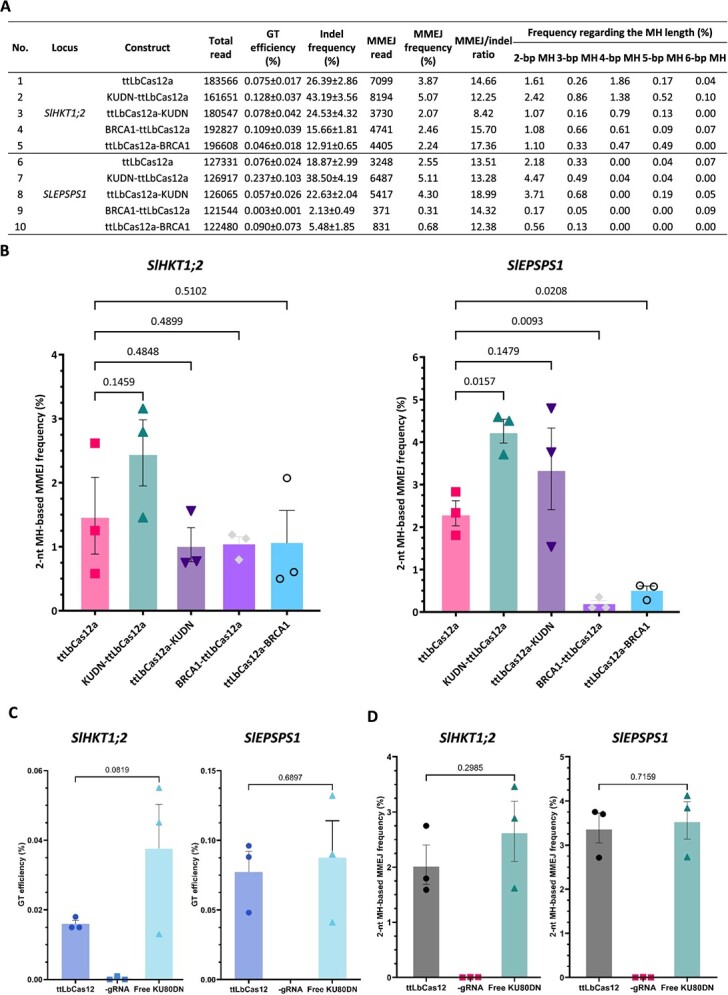
The impacts of KUDN and BRCA1 on the intramolecular MMEJ patterns at the *SlHKT1;2* and *SlEPSPS1* sites. **A,** Table summarizing the impacts of microhomology lengths and MMEJ frequencies. **B,** The 2-nt microhomology-based MMEJ frequency. The indel and MMEJ frequencies were measured by targeted NGS. **C–D,** Impacts of free KUDN on GT (**C**) and 2-nt MH MMEJ (**D**) frequency. *P*-values of the *t*-test for pairwise comparisons are indicated on the top of the bars. All data points are shown as dots in the plots in (**B–D**).

The NKUDN improved MMEJ frequency at 1.31- to 2.00-fold at the *SlHKT1;2* and *SlEPSPS1* sites. Also, the usage of 2-nt MHs was increased with NKUDN at both the targeted sites (Fig. 2a and b). Importantly, it was observed that the MMEJ frequency was highest in the construct containing KUDN-ttLbCas12a (as shown in Fig. 2a and b). This indicates that the 2-nt MH-mediated DSB repair was enhanced, resulting in higher indel mutations, and shifted to the HDR choice.

### Free forms of KUDN showed comparable GT performance

We have observed positive effects of KUDN fusions on DSB repair choice toward HDR (Fig. 1 and 2). We then explored whether similar or more pronounced effects could be achieved using free-form KUDN. Our experiments showed a slight improvement without significance in the GT efficiency at the *SlHKT1;2* site and a comparable performance at the *SlEPSPS1* when KUDN was overexpressed alongside the GT tools (Fig. 2c). Additionally, we found that the GT performance was correlated with the efficiency of indel mutation and MMEJ (Fig. 2d, Fig. S3a–b, and Table S1).

We also used the Suntag system [31] to recruit KUDN to the targeted site. We added 10× Suntag peptide epitopes (GCN4_v4) to the C-terminal of ttLbCas12a and expressed a fusion of scFv-KUDN in parallel (Supplemental sequences). However, the recruitment system unexpectedly reduced GT efficiency at both targeted sites (Fig. S3c and Table S2), possibly due to a correlated reduction of indel mutation efficiency (Fig. S3d).

### BRCA1 fusions to ttLbCas12a reduced the editing activity of the GT tools

BRCA1 plays a crucial role in the HR pathway by interfering with 53BP1 accumulation at the DSB site, enabling end resection and redirecting the repair pathway choice toward HR [32]. Although putative BRCA1 homologs exist in plants, their functional roles are not fully understood yet [33]. In this study, we identified a tomato homolog (Solyc08g023280) of the AtBRCA1 protein and employed it to engineer GT tools to carry BRCA1 to targeted sites from the N- (NBRCA1) or C-terminal (CBRCA1) of ttLbCas12a (Fig. S1b). We observed reduced GT and indel mutation efficiencies with the BRCA1-based GT tools at both targeted sites (Fig. 1a and b) and weaker MMEJ patterns (Fig. 2a and b). Moreover, the GT, indel, and MMEJ frequency was dramatically reduced at the *SlEPSPS1* site (Fig. 1b–c and 2a–b). Surprisingly, though the BRCA1 fusions showed undetectable protein levels (Fig. 1d), the levels of GT at the *SlHKT1;2* site were comparable to the control, indicating a possible stimulating role of BRCA1 in the GT reaction.

### NKUDN enhanced the GT efficiency at the plant stage

**Figure 3:**
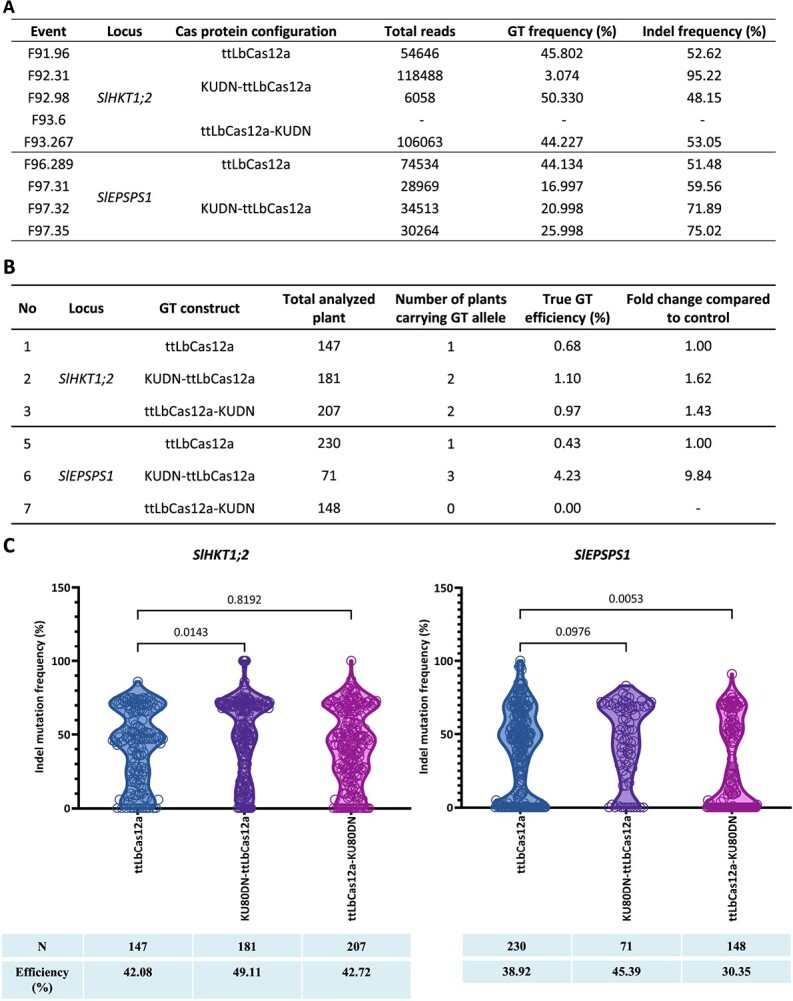
KUDN improved GT efficiency at the plant stage. **A,** Validation of GT0 events by targeted deep sequencing. **B,** Data showing the GT tools’ efficiency at the plant stage using KUDN and the control. **C,** The indel mutation frequency and efficiency of the constructs without and with KUDN fusions. The indel mutation frequency of each transformant is shown as a data point in the violin plots. The statistical analysis was performed using GraphPad 9, and the *P*-values of the pairwise comparisons are shown at the top. The bottom panel summarizes the number of analyzed transformants (N) and the average indel mutation frequency obtained by the constructs. All data points are shown as dots on the plots in (**C**).

An effective GT tool for plant breeding hinges on obtaining edited plants that carry GT alleles with a frequency that ensures their stable inheritance in the next generation. To accurately assess the performance of the KUDN addition to GT tools, we need to determine GT efficiency at the plant stage. We screened the plants obtained from cotyledon explants transformed with the GT constructs for the presence of GT alleles and GT efficiency using a series of methods (Fig. S4). With the GT constructs for editing the *SlHKT1;2* locus, we obtained five GT0 events (F91.96, F92.31, F92.98, F93.6, and F93.267) (Fig. 3a and Fig. S5–S7) out of 535 analyzed transformants. Two of these events were from each construct with KUDN, which proved to be 1.43–1.62 times more efficient than the control construct without KUDN (as shown in Fig. 3a, b). Additionally, NKUDN was found to be the best construct for GT at the *SlEPSPS1* locus, resulting in a 4.23% GT efficiency compared to only 0.43% in the control construct, which is a 9.84-fold enhancement (Fig. 3a–b and Fig. S8, S9). One (F96.289) and three (F97.31, F97.32, and F97.35) *SlEPSPS1* events were obtained using the control and NKUDN-based GT tools, respectively. The data from plant stage analysis again indicate that NKUDN positively impacted GT in tomatoes.

After screening the transformants obtained from the tissue culture of the explants transformed with the GT constructs, it was observed that the construct using NKUDN showed higher indel mutation efficiency and frequency of indel mutation within each plant, as depicted in Fig. 3c. On the other hand, the CKUDN construct showed a comparable efficiency at the *SlHKT1;2* site, while a decrease was observed at the *SlEPSPS1* site. This data is consistent with the results obtained through targeted NGS at the callus stage (Fig. 1).

### KUDN-based GT tools show promise for broad gene targeting in tomato

We experimented to investigate whether the KUDN-based GT tools could be used to introduce specific DNA changes to other parts of the tomato genome. We tested the GT tools to insert a 9-bp DNA sequence into *SlCAB13* (Fig. S10). At the plant stage, we screened 27 transformants and found one precisely edited CAB13 GT0 event (F186NL14, 14.2% GT frequency) (Fig. S11). These results suggest that the KUDN-based GT tools could be used for other parts of the tomato genome, but their efficiency needs to be improved.

### The GT alleles were stably inherited in the next generations.

**Figure 4:**
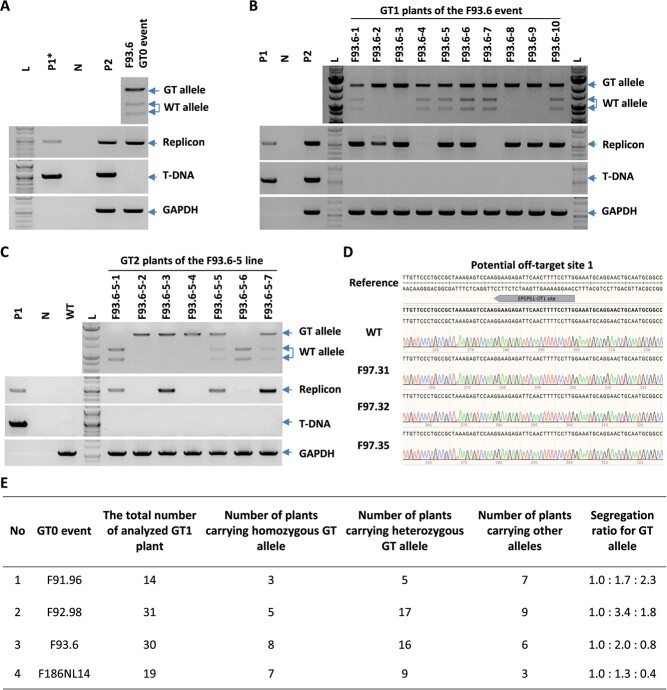
The inheritance of *SlHKT1;2* GT allele in GT1 generation. **A–C,** CAPS assay revealed the GT allele and the presence of T-DNA and replicon in GT0 event F93.6 (**A**), its next generation GT1, (**B**), and GT2 (**C**). **D,** Chromatograms of the sequenced data show no editing traces at the potential off-target sites of *SlEPSPS1* gRNAs. **E,** Table showing the segregation ratios of the GT alleles from four representative GT0 events. F91.96: *SlHKT1;2* GT0 event generated by the GT tool with ttLbCas12a; F92.98: *SlHKT1;2* GT0 event generated by the GT tool with KUDN-ttLbCas12a; F93.6: *SlHKT1;2* GT0 event generated by the GT tool with KUDN-ttLbCas12a; F97.31, F97.32, and F97.35: EPSPS1 GT0 events generated by the GT tool with KUDN-ttLbCas12a; F168NL14: *SlCAB13* GT0 event generated by the GT tool with ttLbCas12a-KUDN.

The GT tools' validity depends on the GT alleles' stable inheritance from GT0 events to their progenies. To confirm this, we raised the next generations of the GT0 events and studied the inheritance of the GT alleles up to GT2. Our analysis showed that all the GT alleles carried by the GT0 events were stably inherited up to GT1 and GT2 generation (as shown in Fig. 4 and Fig. S12), thus confirming the reliability of the KUDN-based GT tools. However, the *SlEPSPS1* GT0 events were too weak to produce fruits and seeds for harvesting. This was likely due to the high levels of *SlEPSPS1* indel frequency (as seen in Fig. S9) carried by the events, leading to insufficient activity of the *SlEPSPS1* enzyme that produces precursors of aromatic amino acids in the shikimate pathway [34]. The weakening of the shikimate pathway could result in reduced growth and fruiting of the edited tomato.

### Obtaining T-DNA and replicon-free GT events was possible via genetic segregation

Obtaining genome-edited plants without containing transgenes is the first demand to be accepted by public legislation before being considered for further steps in the commercialization of the edited plants [35–38]. Our GT system employed a geminiviral replicon for delivering and amplifying GT tools to the targeted sites, thereby enhancing GT efficiency [6]. We previously showed the possibility of obtaining T-DNA and replicon-free GT plants in tomato's next generation of GT0 events [6]. Here, we also assess the presence of T-DNA and replicon in the GT0 events and their progenies.

In the GT0 generation, all events carried either T-DNA or replicon (Fig. S13a). However, we were able to obtain T-DNA and replicon-free plants in F93.6 GT1 (Fig. 4b) and GT2 lines (Fig. 4c) generations.

Similarly, for the *SlCAB13* event, we observed that the GT1 line F168NL-14-9 had a very thin band of replicon and T-DNA (Fig. S13b). However, in the GT2 plants from this line, we obtained all plants that were free of T-DNA, and only some plants showed a band of replicon (Fig. S14). Consequently, we have successfully obtained many homozygous plants that are free of both T-DNA and replicon in GT2 plants such as F168NL14–9-(2,4,5,6,7,8,9,11,12,13,15,16,17) (Fig. S14).

### The KUDN-based GT tools maintained ttLbCas12a specificity without any detected off-target traces

The CRISPR-LbCas12a system uses a 20- to 24-nt gRNA to guide the process. However, this gRNA may bind to unwanted sites adjacent to a T-rich PAM, which can activate the cleavage by LbCas12a and result in off-target indel mutations [39]. Cas12a proteins are highly specific due to their sensitivity to mismatches within the gRNA binding sequence [40]. In plants, LbCas12a and its orthologs have shown high accuracy in editing [6, 41, 42]. The ttLbCas12a used in this study was also highly specific in tomato [6, 13].

To test if the KUDN-based GT tool can alter the specificity of ttLbCas12a, we analyzed and identified potential off-target sites of the used gRNAs by Cas-offinder [43] with less than four mismatches. We found that only some gRNAs that targeted *SlEPSPS1* and *SlCAB13* loci contained several potential off-target sites (Fig. S15a). We amplified and sequenced the sequences, flanking the identified potential off-target sites in the GT events (Fig. S15b). However, no off-target trace was found at all the potential off-targets (Fig. 4d and Fig. S15c). This indicates that the KUDN-based GT tools are highly specific, and adding KUDN did not alter the specificity of ttLbCas12a.

## Discussion

Among the CRISPR-Cas-based approaches, CRISPR-Cas-based GT is the only technique that allows for precise gene/allele replacement at large scales or complex edits, which has not yet been achievable with other precise methods like prime editing [1, 3]. We have developed a geminiviral replicon and ttLbCas12a-based GT system that achieved efficient gene insertion using a double selection method [6]. However, the GT efficiency remains low without using a target-associated selection marker [6, 13] and requires improvement for practical applications.

The low GT efficiency is widely conservative from animals to plants and has a common reason: the cNHEJ is dominant over its competitive HDR pathway for repairing DSBs. Multiple approaches have been successfully employed to enhance HR in animals, and these could be borrowed to enhance GT in plants. In this work, we sought to study the approaches for GT improvement by biasing the DSB repair pathway choice toward HR using the KUDN. We found that directly disturbing the initiation of cNHEJ using KUDN in the second strategy led to a significant enhancement of GT efficiency at the plant stage. We reasoned that the interference of cNHEJ by KUDN increases the activation of the HDR pathway, thereby enhancing GT efficiency.

The analysis of the targeted NGS data during the callus stage showed that the NKUDN enhanced GT efficiency from 1.71- to 3.55-fold in the tested sites (Fig. 1). The enhancing effects of NKUDN were also validated in the plants at both loci, where NKUDN had a 1.62-fold (*SlHKT1;2*) and 9.84-fold (*SlEPSPS1*) enhancement compared to the control (Fig. 3), correlated with the enhancement observed in the *in vitro* samples (Fig. 1b). This data suggests that NKUDN increased the number of cells carrying the GT alleles, leading to a higher chance of obtaining regenerated plants with the GT alleles. It is worth noting that the GT efficiency obtained during the plant stage is more reliable than that of the callus stage, thereby validating the positive impacts of NKUDN for GT in tomatoes. This observation possibly reflects a reduction in cNHEJ repair of DSBs due to the malfunctioning of KU complexes in tomato cells that is consistent with the pioneering work in CHO cells [27] or in *Arabidopsis* [25, 44]. Moreover, the GT enhancement is similar to what was observed in plants with a *ku70*-knockout background [14, 17].

The efficiency of GT can be significantly increased by introducing a DSB at the targeted site [45]. The CRISPR-Cas system utilizes a gRNA-Cas protein complex to introduce DSBs to targeted sites, thereby enhancing GT efficiency [1, 46, 47]. Therefore, the formation of DSBs correlated with the expression levels of the Cas protein, which may affect GT frequency. However, since we observed no significant improvement in protein accumulation between NKUDN and control (Fig. 1d), the enhancement of GT efficiency could be due to the antagonist role of NKUDN toward the KU complex that redirected the repair choice from the cNHEJ route to MMEJ and HR.

When the process of cNHEJ malfunctions, it can redirect the repair of double-strand breaks to the HDR pathway, which includes MMEJ, SSA, and HR [14, 17, 29]. These subpathways require different levels of end resections at varying speeds of repair kinetics [24, 48, 49]. The deletion of short DNA sequences at targeted sites through MMEJ was observed to shift in the editing frequencies (Fig. 2). The use of KUDN constructs led to an increase in MMEJ frequency at the tested loci, as previously recorded by Qi and colleagues in a *ku70*-deficient background [17]. The impact on MMEJ was more pronounced with NKUDN, which was correlated with the enhancement of GT efficiency since the common end resection step initiated both subpathways. The MMEJ traces extracted from targeted NGS data were intrachromosomal repaired products, providing more direct evidence of the KUDN-induced suppression of cNHEJ and subsequent redirection of the DSB repair to the HDR pathway. As a result, the enhancement of MMEJ and other alternative DSB repair pathways that are more error-prone led to an overall increase in the indel mutation efficiency of the NKUDN-based GT tools at both the callus and plant stages (as shown in Fig. 1b and Fig. 3c, respectively). The enhancement of MMEJ frequency by the KUDN may be an interesting feature to improve the chromosome engineering approaches such as inversion [26] or translocation [50].

One of the most important considerations when assessing a gene editing technique is whether the editing tool can be removed from the edited plant or integrated into the plant's genome stably. This is the first criterion for determining whether the edited product is subject to strict or relaxed regulation [51, 52]. In this study, we found that all GT0 events contained either T-DNA or replicon of both cargoes (Fig. 4a and Fig. S13a), but we were able to isolate T-DNA-free and replicon-free GT1 (Fig. 4b) and GT2 (Fig. 4c and Fig. S14) lines of F93.6 and F168NL14 GT0 events by genetically segregating the integrated T-DNAs and replicons (Fig. 4a and Fig. S13a). The replicons existed in a circularized form that did not stably integrate into the genome and could potentially be segregated or diluted out during sexual reproduction (Fig. 4b–c and Fig. S14). It is well known that the replication of viral genomes is largely suppressed in germline cells by systemic RNAi-based gene silencing [53–55]. It is important to note that the efficient transmission of geminiviral genomes through seeds may be limited to specific strains and has a size constraint [56, 57]. In our study, the reduced seed transmission rate was possibly due to the large size of the GT tool-carrying circularized replicons engineered from a BeYDV [6]. More comprehensive analysis, such as in the case of the GABA tomato [36], using multiple primer pairs covering the entire plasmid sequence for polymerase chain reaction (PCR) or whole-genome sequencing, should be performed upon the commercialization of a GT plant.

Importantly, the GT tools did not induce off-targeting activities in other genome sites (Fig. S15). The T-rich PAM LbCas12a is more specific than SpCas9 due to its more extended PAM sequence and hypersensitivity toward mismatches within the binding sequence of gRNAs [6, 40, 42, 58].

To summarize, our data support the hypothesis that adding KUDN interferes with cNHEJ, shifting DSB repair to HDR subpathways, and ultimately enhancing GT efficiency.

## Materials and methods

### System design and plasmid cloning

Initially, the tomato *HKT1;2* and *EPSPS1* loci were selected as targets for conducting HR-based allele replacement experiments since they were studied in our lab [6, 13]. Subsequently, *SlCAB13* was used to show the applicability of the improved KUDN-carrying GT tool. *SlCAB13* is a gene encoding for a light-harvesting chlorophyll a/b binding protein (type III, homolog 13) that plays essential roles in photosynthesis. Missing AAATTGTGA proximal to the start codon of *SlCAB13* in domesticated tomato led to sensitivity to continuous lighting conditions [59]. We designed the GT construct to restore the bases to the corresponding location of the *SlCAB13* gene (AAATTGTGA insertion) that may help tomato tolerate continuous lighting conditions, thereby increasing tomato yield. The homologous donor for targeting *SlCAB13* and the gRNA expression cassette were designed and cloned accordingly, as shown in Fig. S10 and the Supplemental sequence file.

A geminiviral replicon system was employed as the vector for the delivery of guide RNA and CRISPR-Cas expression cassettes, as well as GT donor templates (Fig. 1a and Fig. S2) of all the constructs. A dual gRNA construct was employed for each of the targeted sites, which were designed in tandem repeats of LbCas12a scaffolds and gRNA sequences (Fig. S2a). The loci and gRNA sequences are listed in Table S3. The plasmids containing fusions (KUDN, BRCA1, expression cassettes, and the binary vectors) (Fig. S2) were cloned using the Golden Gate assembly method [60, 61]. The DNA sequences are listed in the Supplemental sequence file. Vu and coworkers designed the homologous donors of the *SlHKT1;2* and *SlEPSPS1* earlier [13].

All the Cas protein expression cassettes used the long CaMV35S long promoter (Addgene #50267) and CaMV 35S terminator (Addgene #50337). An additional copy of the *AtTRP1* intron 1 was inserted into the coding sequence of the ttLbCas12a variant, a modification previously tested in GT experiments by Vu and colleagues [6, 13]. The crRNA and sgRNA expression cassettes were transcribed using the core sequence of the AtU6 promoter [62] and terminated by an oligo dT (7xT).

### *Agrobacterium*-mediated tomato transformation

For our study, we used a local tomato cultivar called Hongkwang and performed *Agrobacterium*-mediated tomato transformation following the established protocol of our laboratory [6]. First, *Agrobacterium tumefaciens* GV3101::pMP90 cells were cultured overnight in primary culture using LB medium with appropriate antibiotics in a shaking incubator at 30°C. The agrobacterium cells were then harvested from the culture by centrifugation and suspended in a liquid ABM-MS medium (pH 5.2) with AS (200 μM). The OD600nm of the suspension was adjusted to 0.8. Cotyledon fragments were cut from 7-day-old seedlings and precultured on the PREMC medium (containing MS salts, B5 vitamins, 2 mg/l zeatin trans-isomer, 0.5 mg/l IAA, 1 mM putrescine, 0.5 mg/l MES, 30 g/l maltose, and 7.5 g/l agar) 1 day before the transformation. The transformation process involved mixing tomato cotyledon fragments with the bacterium suspension and keeping them at room temperature for 25 min. The explants were then transferred to the cocultivation medium containing all the elements from the ABM-MS medium and AS (200 μM) at pH 5.8. These co-cultivation plates were placed in darkness at 25°C for 2 days, and the explants were moved to a non-selection medium (NSELN) for 5 days before being subcultured into the selection medium (SEL5).

The NSELN and SEL5 media contained all the components of the preculture medium, supplemented with 250 mg/l timentin and 60 mg/l kanamycin. NSELN also contained 2 μM NU7441. The explants were subsequently subcultured on SEL5R (SEL5 with 1 mg/l zeatin trans-isomer), SEL4CA (SEL5 with 0.5 mg/l zeatin trans-isomer, 0.05 mf/l IAA, 0.1 g/l ascorbic acid, and 5 mg/l AgNO3), and SEL4C (SEL4CA without AgNO3) every 15 days to improve regeneration efficiency. Once the shoots grew to a length of ~2.0 cm, they were subcultured to the RIM medium for rooting. The RIM medium was similar to SEL4C but without zeatin trans-isomer and IAA and contained 0.1 mg/l NAA and 0.3 mg/l IBA. The shoots with roots were acclimated in a greenhouse under 16 h of light and 8 h of darkness at a temperature of 26 ± 2°C.

### Plant genomic isolation by CTAB method

To extract the genomic DNA (gDNA) from plant leaves or cotyledon explants/callus, we used the CTAB method with some minor modifications based on Vu and coworkers [13]. We started by grinding ~200 mg of leaf sample in liquid nitrogen and adding 300 μl of Solution I, which contained 1 M NaCl, 2% Sarkosyl, and 5 μl of RNase at 10 mg/ml/tube. The mixture was then incubated at 37°C for 30 min and centrifuged at 13 000 rpm at 4°C for 10 min. We then incubated 250 μl of the supernatant with 500 μl of extraction buffer (containing 100 mM Tris-Cl, 20 mM EDTA, 1.4 M NaCl, and 2% CTAB) at 60°C for 35 min. After that, we added 750 μl of Chloroform:Isoamylalcohol (24:1) and centrifuged the mixture at 13 000 rpm at 4°C for 15 min. We added 20 μl of 3 M CH3COONa (pH 5.2) and 360 μl of isopropanol to the 600 μl supernatant liquid, followed by centrifugation at 13 000 rpm at 4°C for 5 min. The genomic DNA (pellet) was washed with 80% ethanol and centrifuged again at 13 000 rpm at 4°C for 5 min. Finally, we incubated the DNA at 37°C for 30 min to remove residual ethanol and dissolved the gDNA pellet in 50 μl of DNA Elution buffer (EB). We then used a NanoDrop 1000 UV/Vis Spectrophotometer (NanoDrop Technologies Inc., Wilmington, DE, USA) to determine the quality and concentration of the DNA.

### Targeted deep sequencing

The method used for targeted NGS analysis was previously described by Vu and coworkers [13] and was slightly modified for this study. PCR was used to amplify the targeted sites in the gDNA isolated from the cotyledon/callus samples, using the first PCR primer pair (Table S4), which was located outside the homologous sequences and flanking the editing sites. The second and third PCR reactions were carried out according to the guidelines provided by the miniseq sequencing service provider (KAIST Bio-Core Center, Daejeon, Korea). The raw data files were preprocessed by the NGS service provider and were subsequently analyzed using the RGEN Cas-analyzer tool [63] and CRISPResso2 [64]. The targeted deep sequencing of the first batch of the experiment (end resection facilitation) was conducted with 10-dpt samples, as reported earlier [13]. However, since the GT efficiency was low and highly variable, we used 21-dpt samples for the analysis afterward.

At the callus stage, GT efficiency is calculated as follows: GT(%) = 100 × (number of NGS reads containing GT alleles/total NGS reads).

### Validation of GT events by molecular analyses

The CTAB method was used to extract genomic DNAs from tomato. PCR was used to verify the GT sites and donor junctions using the first primer pairs that covered the respective sites (Table S4). High-fidelity Taq DNA polymerase (Phusion Taq, Thermo Fisher Scientific, Massachusetts, USA) was employed in this process, followed by Sanger sequencing (Solgent, Daejeon, Korea) for analysis. To differentiate GT-edited events due to changes in the cutting site of enzymes during donor design and cloning, the cleaved amplified polymorphic sequence (CAPS) method was used. BpiI restriction enzyme was used to digest PCR products. The binding site of BpiI was modified in the GT alleles. BpiI did not digest potential GT products, and the PCR products containing the undigested band were sent for Sanger sequencing. Furthermore, the ICE-Synthego program was used to decompose the Sanger sequencing results, which helped identify indel mutations and GT efficiency within the plant. We then assessed the potential GT events that showed GT allele frequency >10% in ICE Synthego data by targeted NGS to validate whether they were true GT events. Their PCR products were cloned into pJET1.2 blunt plasmids (Thermo Fisher Scientific, Massachusetts, USA) and sequenced.

GT efficiency at the plant stage was calculated as GT(%) = 100*(Total plants carrying GT alleles/Total analyzed plants).

PCR reactions were performed using the primers listed in Table S4 to determine whether GT events contained T-DNA and replicon. The protocol provided by the manufacturer of Diastar Taq DNA polymerase (Solgent, Daejeon, Korea) was followed, and the PCR reactions were run for 30 cycles. The resulting PCR products were then separated on a 0.8% agarose gel. The presence or absence of T-DNA or replicon was determined based on the presence or absence of DNA bands on the agarose gels.

### Western blot analysis

Tobacco leaves that were 35 days old were infiltrated with Agrobacterium clones containing the GT constructs. After 3 days, an equal amount of infiltrated tobacco leaf samples was collected and kept in liquid nitrogen for grinding. Total proteins were extracted using a previously reported protocol [65]. The extracted proteins (~40 μg) were loaded onto 5% SDS-PAGE gels with a PM2610 protein ladder (SMOBIO, Hsinchu, TW), and electrophoresis was run at a fixed 100 V for 2 h. The proteins were then transferred from the gel to a PVDF membrane (Immonilon-P transfer membrane, Merck, Darmstadt, Germany) using the wet transfer method, which was conducted for 2 h with a transfer buffer containing Tris-base, glycine, and 20% methanol. The membrane was blocked using a buffer containing 5% low-fat milk powder in 1x TBS-T (Tris, NaCl, Tween) under gentle shaking conditions (50 rpm) for 2 h. The membrane was then incubated overnight in a 5% low-fat milk powder buffer in 1x TBS-T and HA antibody (1:5000) at 4°C. The β-actin-specific antibody (1:20 000) was used as an internal control. The next day, the membrane was washed five times with 1x TBS-T, and the second antibody (antirabbit, diluted at a 1:10 000 ratio in 1x TBS-T) was added to the membrane. The membrane was incubated at 4°C overnight. The following morning, the membrane was washed twice with the 1x TBS-T buffer and then incubated with the Clarity Western ECL Substrate solution (Bio-Rad, California, USA). Signal detection of the membranes was performed by the Chemi-Doc Imaging system (Bio-Rad, California, USA) after a 5-min incubation. The images were taken every 5 s for 30 s, and the GT constructs' protein expression was quantified using ImageJ software (NIH, Maryland, USA).

### Off-target analysis

To identify any potential off-target locations in the tomato genome, we used the sequence of each gRNA as a query sequence in http://www.rgenome.net/cas-offinder. For this purpose, we selected the PAM type of LbCpf1 (5’TTTN3’) and the *Solanum lycopersicum* (SL2.4)-Tomato genome. We then designed specific primers for off-target sites using the NCBI PrimerBlast and used them for PCR amplification and Sanger sequencing (as outlined in Table S5) to verify the results.

### Assessment of the inheritance of GT alleles in the next generation of GT0 events

After successfully validating GT0 events, they underwent self-pollination, and the resulting seeds were collected and stored for further investigation. These seeds were then planted in soil, and the genomic DNAs of the offspring plants were individually extracted for examination through Sanger sequencing. The sequencing chromatogram files obtained were subsequently analyzed with the ICE Synthego tool, which provided useful insights into each plant's GT and indels frequencies. Moreover, PCRs were performed using the primers specified in Table S5S4 to assess the presence of replicon and T-DNA constructs.

### Statistical analysis

All comparison experiments were conducted with at least three replicates, and the data was collected by counting purple spots, performing targeted deep sequencing, and screening plant events. Some of the experiments using targeted deep sequencing were conducted in two replicates. The editing data, statistical analysis, and plots were further processed using MS Excel and GraphPad Prism 9 programs and explained in detail in the legends of Figures and Tables. Pairwise comparison data were tested with Student's *t*-test with unequal variance and two-tailed parameters. Fisher's LSD test was applied for multiple comparisons using similar parameters. A difference was considered significant when the statistical tests returned a *P*-value of <.05.

## Supplementary Material

Web_Material_uhae294

## Data Availability

The data underlying this article are available in the article and in its online supplementary material.
