# Supplementary material for: Enhancing CRISPR-Cas-based gene targeting in tomato using a dominant-negative ku80
Source: Hortic Res. 2024 Oct 23;12(2):uhae294. doi: 10.1093/hr/uhae294 (PMC11789525; doi:10.1093/hr/uhae294)
Supplement: Web_Material_uhae294 [file web_material_uhae294.zip › 24.05.28_Supplemental sequences_tvv1.pdf]

Sequences used in the study

- **ttLbCas12a sequence**

ATGCCCAAGAAGAAGCGCAAGGTGGGACGCGTCTGCAGGATATCAAGCTTGCGGTACCGCGGCCCG
GGATCGCCACCATGAGCAAGCTGGAGAAGTTTACAAACTGCTACTCCCTGTCTAAGACCCTGAGGTTCA
AGGTAAAGCCTCGATTTTTGGGTTTAGGTGTCTGCTTATTAGAGTAAAAACACATCCTTTGAAATTGTTT
GTGGTCATTTGATTGTGCTCTTGATCCATTGAATTGCTGCAGGCCATCCCTGTGGGCAAGACCCAGGAGA
ACATCGACAATAAGCGGCTGCTGGTGGAGGACGAGAAGAGAGCCGAGGATTATAAGGGCGTGAAGAA
GCTGCTGGATCGCTACTATCTGTCTTTTATCAACGACGTGCTGCACAGCATCAAGCTGAAGAATCTGAAC
AATTACATCAGCCTGTTCCGGAAGAAAACCAGAACCGAGAAGGAGAATAAGGAGCTGGAGAACCTGGA
GATCAATCTGCGGAAGGAGATCGCCAAGGCCTTCAAGGGCAACGAGGGCTACAAGTCCCTGTTTAAGA
AGGATATCATCGAGACAATCCTGCCAGAGTTCCTGGACGATAAGGACGAGATCGCCCTGGTGAACAGCT
TCAATGGCTTTACCACAGCCTTCACCGGCTTCTTTAGAAACAGAGAGAATATGTTTTCCGAGGAGGCCAA
GAGCACATCCATCGCCTTCAGGTGTATCAACGAGAATCTGACCCGCTACATCTCTAATATGGACATCTTC
GAGAAGGTGGACGCCATCTTTGATAAGCACGAGGTGCAGGAGATCAAGGAGAAGATCCTGAACAGCG
ACTATGATGTGGAGGATTTCTTTGAGGGCGAGTTCTTTAACTTTGTGCTGACACAGGAGGGCATCGACG
TGTATAACGCCATCATCGGCGGCTTCGTGACCGAGAGCGGCGAGAAGATCAAGGGCCTGAACGAGTAC
ATCAACCTGTATAATCAGAAAACCAAGCAGAAGCTGCCTAAGTTTAAGCCACTGTATAAGCAGGTGCTG
AGCGATCGGGAGTCTCTGAGCTTCTACGGCGAGGGCTATACATCCGATGAGGAGGTGCTGGAGGTGTT
TAGAAACACCCTGAACAAGAACAGCGAGATCTTCAGCTCCATCAAGAAGCTGGAGAAGCTGTTCAAGAA
TTTTGACGAGTACTCTAGCGCCGGCATCTTTGTGAAGAACGGCCCCGCCATCAGCACAATCTCCAAGGAT
ATCTTCGGCGAGTGGAACGTGATCCGGGACAAGTGGAATGCCGAGTATGACGATATCCACCTGAAGAA
GAAGGCCGTGGTGACCGAGAAGTACGAGGACGATCGGAGAAAGTCCTTCAAGAAGATCGGCTCCTTTT
CTCTGGAGCAGCTGCAGGAGTACGCCGACGCCGATCTGTCTGTGGTGGAGAAGCTGAAGGAGATCATC
ATCCAGAAGGTGGATGAGATCTACAAGGTGTATGGCTCCTCTGAGAAGCTGTTCGACGCCGATTTTGTG
CTGGAGAAGAGCCTGAAGAAGAACGACGCCGTGGTGGCCATCATGAAGGACCTGCTGGATTCTGTGAA
GAGCTTCGAGAATTACATCAAGGCCTTCTTTGGCGAGGGCAAGGAGACAAACAGGGACGAGTCCTTCT
ATGGCGATTTTGTGCTGGCCTACGACATCCTGCTGAAGGTGGACCACATCTACGATGCCATCCGCAATTA
TGTGACCCAGAAGCCCTACTCTAAGGATAAGTTCAAGCTGTATTTTCAGAACCCTCAGTTCATGGGCGGC
TGGGACAAGGATAAGGAGACAGACTATCGGGCCACCATCCTGAGATACGGCTCCAAGTACTATCTGGC
CATCATGGATAAGAAGTACGCCAAGTGCCTGCAGAAGATCGACAAGGACGATGTGAACGGCAATTACG
AGAAGATCAACTATAAGCTGCTGCCCGGCCCTAATAAGATGCTGCCAAAGGTGTTCTTTTCTAAGAAGT
GGATGGCCTACTATAACCCCAGCGAGGACATCCAGAAGATCTACAAGAATGGCACATTCAAGAAGGGC
GATATGTTTAACCTGAATGACTGTCACAAGCTGATCGACTTCTTTAAGGATAGCATCTCCCGGTATCCAA
AGTGGTCCAATGCCTACGATTTCAACTTTTCTGAGACAGAGAAGTATAAGGACATCGCCGGCTTTTACAG
AGAGGTGGAGGAGCAGGGCTATAAGGTGAGCTTCGAGTCTGCCAGCAAGAAGGAGGTGGATAAGCTG
GTGGAGGAGGGCAAGCTGTATATGTTCCAGATCTATAACAAGGACTTTTCCGATAAGTCTCACGGCACA
CCCAATCTGCACACCATGTACTTCAAGCTGCTGTTTGACGAGAACAATCACGGACAGATCAGGCTGAGC

GGAGGAGCAGAGCTGTTCATGAGGCGCGCCTCCCTGAAGAAGGAGGAGCTGGTGGTGCACCCAGCCA
ACTCCCCTATCGCCAACAAGAATCCAGATAATCCCAAGAAAACCACAACCCTGTCCTACGACGTGTATAA
GGATAAGAGGTTTTCTGAGGACCAGTACGAGCTGCACATCCCAATCGCCATCAATAAGTGCCCCAAGAA
CATCTTCAAGATCAATACAGAGGTGCGCGTGCTGCTGAAGCACGACGATAACCCCTATGTGATCGGCAT
CGATAGGGGCGAGCGCAATCTGCTGTATATCGTGGTGGTGGACGGCAAGGGCAACATCGTGGAGCAGT
ATTCCCTGAACGAGATCATCAACAACTTCAACGGCATCAGGATCAAGACAGATTACCACTCTCTGCTGGA
CAAGAAGGAGAAGGAGAGGTTCGAGGCCCGCCAGAACTGGACCTCCATCGAGAATATCAAGGAGCTG
AAGGCCGGCTATATCTCTCAGGTGGTGCACAAGATCTGCGAGCTGGTGGAGAAGTACGATGCCGTGAT
CGCCCTGGAGGACCTGAACTCTGGCTTTAAGAATAGCCGCGTGAAGGTGGAGAAGCAGGTGTATCAGA
AGTTCGAGAAGATGCTGATCGATAAGCTGAACTACATGGTGGACAAGAAGTCTAATCCTTGTGCAACAG
GCGGCGCCCTGAAGGGCTATCAGATCACCAATAAGTTCGAGAGCTTTAAGTCCATGTCTACCCAGAACG
GCTTCATCTTTTACATCCCTGCCTGGCTGACATCCAAGATCGATCCATCTACCGGCTTTGTGAACCTGCTG
AAAACCAAGTATACCAGCATCGCCGATTCCAAGAAGTTCATCAGCTCCTTTGACAGGATCATGTACGTGC
CCGAGGAGGATCTGTTCGAGTTTGCCCTGGACTATAAGAACTTCTCTCGCACAGACGCCGATTACATCAA
GAAGTGGAAGCTGTACTCCTACGGCAACCGGATCAGAATCTTCCGGAATCCTAAGAAGAACAACGTGTT
CGACTGGGAGGAGGTGTGCCTGACCAGCGCCTATAAGGAGCTGTTCAACAAGTACGGCATCAATTATCA
GCAGGGCGATATCAGAGCCCTGCTGTGCGAGCAGTCCGACAAGGCCTTCTACTCTAGCTTTATGGCCCT
GATGAGCCTGATGCTGCAGATGCGGAACAGCATCACAGGCCGCACCGACGTGGATTTTCTGATCAGCCC
TGTGAAGAACTCCGACGGCATCTTCTACGATAGCCGGAACTATGAGGCCCAGGAGAATGCCATCCTGCC
AAAGAACGCCGACGCCAATGGCGCCTATAACATCGCCAGAAAGGTGCTGTGGGCCATCGGCCAGTTCA
AGAAGGCCGAGGACGAGAAGCTGGATAAGGTGAAGATCGCCATCTCTAACAAGGAGTGGCTGGAGTA
CGCCCAGACCAGCGTGAAGCACGCCTATCCCTATGACGTGCCCGATTATGCCAGCCTGGGCAGCGGCTC
CCCCAAGAAAAAACGCAAGGTGGAAGATCCTAAGAAAAAGCGGAAAGTGGACGGCATTGGTAGTGGG
AGCTAAGCTT

Purple font: SV40 NLS; blue font: LbCas12a; 1xHA tag; tan font: Trp1 intron; yellow highlighted font: D156R modification of the temperature tolerant LbCas12a; black font: linkers.

- **ttLbCas12a-10xSuntag sequence**

ATGCCCAAGAAGAAGCGCAAGGTGGGACGCGTCTGCAGGATATCAAGCTTGCGGTACCGCGGGCCCG
GGATCGCCACCATGAGCAAGCTGGAGAAGTTTACAAACTGCTACTCCCTGTCTAAGACCCTGAGGTTCA
AGGTAAAGCCTCGATTTTTGGGTTTAGGTGTCTGCTTATTAGAGTAAAAACACATCCTTTGAAATTGTTT
GTGGTCATTTGATTGTGCTCTTGATCCATTGAATTGCTGCAGGCCATCCCTGTGGGCAAGACCCAGGAGA
ACATCGACAATAAGCGGCTGCTGGTGGAGGACGAGAAGAGAGCCGAGGATTATAAGGGCGTGAAGAA
GCTGCTGGATCGCTACTATCTGTCTTTTATCAACGACGTGCTGCACAGCATCAAGCTGAAGAATCTGAAC
AATTACATCAGCCTGTTCCGGAAGAAAACCAGAACCGAGAAGGAGAATAAGGAGCTGGAGAACCTGGA
GATCAATCTGCGGAAGGAGATCGCCAAGGCCTTCAAGGGCAACGAGGGCTACAAGTCCCTGTTTAAGA
AGGATATCATCGAGACAATCCTGCCAGAGTTCCTGGACGATAAGGACGAGATCGCCCTGGTGAACAGCT
TCAATGGCTTTACCACAGCCTTCACCGGCTTCTTTAGAAACAGAGAGAATATGTTTTCCGAGGAGGCCAA
GAGCACATCCATCGCCTTCAGGTGTATCAACGAGAATCTGACCCGCTACATCTCTAATATGGACATCTTC

GAGAAGGTGGACGCCATCTTTGATAAGCACGAGGTGCAGGAGATCAAGGAGAAGATCCTGAACAGCG
ACTATGATGTGGAGGATTTCTTTGAGGGCGAGTTCTTTAACTTTGTGCTGACACAGGAGGGCATCGACG
TGTATAACGCCATCATCGGCGGCTTCGTGACCGAGAGCGGCGAGAAGATCAAGGGCCTGAACGAGTAC
ATCAACCTGTATAATCAGAAAACCAAGCAGAAGCTGCCTAAGTTTAAGCCACTGTATAAGCAGGTGCTG
AGCGATCGGGAGTCTCTGAGCTTCTACGGCGAGGGCTATACATCCGATGAGGAGGTGCTGGAGGTGTT
TAGAAACACCCTGAACAAGAACAGCGAGATCTTCAGCTCCATCAAGAAGCTGGAGAAGCTGTTCAAGAA
TTTTGACGAGTACTCTAGCGCCGGCATCTTTGTGAAGAACGGCCCCGCCATCAGCACAATCTCCAAGGAT
ATCTTCGGCGAGTGGAACGTGATCCGGGACAAGTGGAATGCCGAGTATGACGATATCCACCTGAAGAA
GAAGGCCGTGGTGACCGAGAAGTACGAGGACGATCGGAGAAAGTCCTTCAAGAAGATCGGCTCCTTTT
CTCTGGAGCAGCTGCAGGAGTACGCCGACGCCGATCTGTCTGTGGTGGAGAAGCTGAAGGAGATCATC
ATCCAGAAGGTGGATGAGATCTACAAGGTGTATGGCTCCTCTGAGAAGCTGTTCGACGCCGATTTTGTG
CTGGAGAAGAGCCTGAAGAAGAACGACGCCGTGGTGGCCATCATGAAGGACCTGCTGGATTCTGTGAA
GAGCTTCGAGAATTACATCAAGGCCTTCTTTGGCGAGGGCAAGGAGACAAACAGGGACGAGTCCTTCT
ATGGCGATTTTGTGCTGGCCTACGACATCCTGCTGAAGGTGGACCACATCTACGATGCCATCCGCAATTA
TGTGACCCAGAAGCCCTACTCTAAGGATAAGTTCAAGCTGTATTTTCAGAACCCTCAGTTCATGGGCGGC
TGGGACAAGGATAAGGAGACAGACTATCGGGCCACCATCCTGAGATACGGCTCCAAGTACTATCTGGC
CATCATGGATAAGAAGTACGCCAAGTGCCTGCAGAAGATCGACAAGGACGATGTGAACGGCAATTACG
AGAAGATCAACTATAAGCTGCTGCCCGGCCCTAATAAGATGCTGCCAAAGGTGTTCTTTTCTAAGAAGT
GGATGGCCTACTATAACCCCAGCGAGGACATCCAGAAGATCTACAAGAATGGCACATTCAAGAAGGGC
GATATGTTTAACCTGAATGACTGTCACAAGCTGATCGACTTCTTTAAGGATAGCATCTCCCGGTATCCAA
AGTGGTCCAATGCCTACGATTTCAACTTTTCTGAGACAGAGAAGTATAAGGACATCGCCGGCTTTTACAG
AGAGGTGGAGGAGCAGGGCTATAAGGTGAGCTTCGAGTCTGCCAGCAAGAAGGAGGTGGATAAGCTG
GTGGAGGAGGGCAAGCTGTATATGTTCCAGATCTATAACAAGGACTTTTCCGATAAGTCTCACGGCACA
CCCAATCTGCACACCATGTACTTCAAGCTGCTGTTTGACGAGAACAATCACGGACAGATCAGGCTGAGC
GGAGGAGCAGAGCTGTTCATGAGGCGCGCCTCCCTGAAGAAGGAGGAGCTGGTGGTGCACCCAGCCA
ACTCCCCTATCGCCAACAAGAATCCAGATAATCCCAAGAAAACCACAACCCTGTCCTACGACGTGTATAA
GGATAAGAGGTTTTCTGAGGACCAGTACGAGCTGCACATCCCAATCGCCATCAATAAGTGCCCCAAGAA
CATCTTCAAGATCAATACAGAGGTGCGCGTGCTGCTGAAGCACGACGATAACCCCTATGTGATCGGCAT
CGATAGGGGCGAGCGCAATCTGCTGTATATCGTGGTGGTGGACGGCAAGGGCAACATCGTGGAGCAGT
ATTCCCTGAACGAGATCATCAACAACTTCAACGGCATCAGGATCAAGACAGATTACCACTCTCTGCTGGA
CAAGAAGGAGAAGGAGAGGTTCGAGGCCCGCCAGAACTGGACCTCCATCGAGAATATCAAGGAGCTG
AAGGCCGGCTATATCTCTCAGGTGGTGCACAAGATCTGCGAGCTGGTGGAGAAGTACGATGCCGTGAT
CGCCCTGGAGGACCTGAACTCTGGCTTTAAGAATAGCCGCGTGAAGGTGGAGAAGCAGGTGTATCAGA
AGTTCGAGAAGATGCTGATCGATAAGCTGAACTACATGGTGGACAAGAAGTCTAATCCTTGTGCAACAG
GCGGCGCCCTGAAGGGCTATCAGATCACCAATAAGTTCGAGAGCTTTAAGTCCATGTCTACCCAGAACG
GCTTCATCTTTTACATCCCTGCCTGGCTGACATCCAAGATCGATCCATCTACCGGCTTTGTGAACCTGCTG
AAAACCAAGTATACCAGCATCGCCGATTCCAAGAAGTTCATCAGCTCCTTTGACAGGATCATGTACGTGC
CCGAGGAGGATCTGTTCGAGTTTGCCCTGGACTATAAGAACTTCTCTCGCACAGACGCCGATTACATCAA
GAAGTGGAAGCTGTACTCCTACGGCAACCGGATCAGAATCTTCCGGAATCCTAAGAAGAACAACGTGTT
CGACTGGGAGGAGGTGTGCCTGACCAGCGCCTATAAGGAGCTGTTCAACAAGTACGGCATCAATTATCA

GCAGGGCGATATCAGAGCCCTGCTGTGCGAGCAGTCCGACAAGGCCTTCTACTCTAGCTTTATGGCCCT
GATGAGCCTGATGCTGCAGATGCGGAACAGCATCACAGGCCGCACCGACGTGGATTTCTGATCAGCCC
TGTGAAGAACTCCGACGGCATCTTCTACGATAGCCGGAACTATGAGGCCCAGGAGAATGCCATCCTGCC
AAAGAACGCCGACGCCAATGGCGCCTATAACATCGCCAGAAAGGTGCTGTGGGCCATCGGCCAGTTCA
AGAAGGCCGAGGACGAGAAGCTGGATAAGGTGAAGATCGCCATCTCTAACAAGGAGTGGCTGGAGTA
CGCCCAGACCAGCGTGAAGCACGCCTATCCCTATGACGTGCCCGATTATGCCAGCCTGGGCAGCGGCTC
CCCCAAGAAAAAACGCAAGGTGGAAGATCCTAAGAAAAAGCGGAAAGTGGACGGCATTGGTAGTGGG
AGCGgaggtgggggatctggttcgatgGAAGAACTTTTGAGCAAGAATTATCATCTTGAGAACGAAGTGGCTCG
TCTTAAGAAAGGTTCTGGCAGTGGAGAAGAACTGCTTTCAAAGAATTACCACCTGGAAAATGAGGTAGC
TAGACTGAAAAAGGGGAGCGGAAGTGGGGAGGAGTTGCTGAGCAAAAATTATCATTTGGAGAACGAA
GTAGCACGACTAAAGAAAGGGTCCGGATCGGGTGAGGAGTTACTCTCGAAAAATTATCATCTCGAAAAC
GAAGTGGCTCGGCTAAAAAAGGGCAGTGGTTCTGGAGAAGAGCTATTATCTAAAAACTACCACCTCGAA
AATGAGGTGGCACGCTTAAAAAAGGGAAGTGGCAGTGGTGAAGAGCTACTATCCAAGAATTATCATCT
TGAGAACGAGGTAGCGCGTTTGAAGAAGGGTTCCGGCTCAGGAGAGGAACTGCTCTCGAAGAACTATC
ATCTTGAAAATGAGGTCGCTCGATTAAAAAAGGGATCGGGCAGTGGTGAGGAACTACTTTCAAAGAATT
ACCACCTCGAAAACGAAGTAGCTCGATTAAAGAAAGGTTCAGGGTCGGGTGAAGAATTACTGAGTAAA
AATTATCATCTGGAAAATGAGGTAGCGAGACTAAAAAAGGGGAGTGGTTCTGGCGAGGAATTGCTATC
GAAAAATTATCATCTTGAGAACGAAGTTGCTAGGCTCAAAAAGGGCTCAGGCTCAGGCACCGCGtaa

Purple font: SV40 NLS; blue font: LbCas12a; 1xHA tag; tan font: Trp1 intron; red font: GCN4_v4 (Suntag); yellow highlighted font: D156R modification of the temperature tolerant LbCas12a; black font: linkers.

❖ ***SlKU80DN* sequence for fusions, cloned using tomato genomic DNA**

AAGCAACAAGACGCAGCAGATAAATTGGTTCAGATGTTGGATCTTGCACCACCTGGAAAACAGGAAGT
GTTATCACCTGACTTCACACCTAATCCTGTTCTAGAGCGTTACTACCGCTATCTTAACCTGAAGTCAAAGC
ACCCAGATGCAGCTGTTCCTCCACTTGATGAAACCCTCAGAAAGATAACAGAACCTGATGTTGAACTTCT
TTCTCAAAACAAGTCCATCATAGAGGAACTCCGTAGGTCTTTTGAACTAAAAGATAATCCAAAGCTGAAA
AAATCAGCAAGAAGAATAAAAGAaAGACCTTCAGGATCAGATGAGGAGATAGAAGAATTCAACAAAGA
TGCTGATGTCAAAGCTATAGACTCCATGGAATACTCAGCCAAAACAGAAGTTGAGAAAGTTGGAGATGT
TAATCCTGTCAAAGACTTTGAGGATATGCTGTCTCGAAGAGATAATCCAAAATGGATTAGTAAGGCCATT
CAGGATATGAAAAATAGGATCTTTGATCTCGTCGAAAATTCTTGTGACGGAGATACATTTCATAAAGCAT
TGCAATGTTTGGTGGCTCTACGCAAAGGTTGCATCCTTGAGCAGGAACCAAAGCAGTTCAATGATTTCCT
GTGCCACCTATCTAAATTTTGCCAAGAAAAAGACCTGAGAAGTTTCTGTCTATATCTCACATCTCATGAAA
TCACTTTGATAACCAAGGCAGAAGCTCCAGACAGTGAAATTTCAGAACATGAGGCTAGAAGCTTTATGG
TCAAGCCTGAACTTGACTCGCAAAATATGAAATCAGAGGCTAAAGCAGAGGATGATATTATGAGTATAT
ACCTGGGAGGGAAG

❖ ***SlKU80DN* sequence for overexpression in free form, cloned using tomato genomic DNA**

ATGAAGCAACAAGACGCAGCAGATAAATTGGTTCAGATGTTGGATCTTGCACCACCTGGAAAACAGGA
AGTGTTATCACCTGACTTCACACCTAATCCTGTTCTAGAGCGTTACTACCGCTATCTTAACCTGAAGTCAA
AGCACCCAGATGCAGCTGTTCCTCCACTTGATGAAACCCTCAGAAAGATAACAGAACCTGATGTTGAACT
TCTTTCTCAAAACAAGTCCATCATAGAGGAACTCCGTAGGTCTTTTGAACTAAAAGATAATCCAAAGCTG
AAAAAATCAGCAAGAAGAATAAAAGAaAGACCTTCAGGATCAGATGAGGAGATAGAAGAATTCAACAA
AGATGCTGATGTCAAAGCTATAGACTCCATGGAATACTCAGCCAAAACAGAAGTTGAGAAAGTTGGAG
ATGTTAATCCTGTCAAAGACTTTGAGGATATGCTGTCTCGAAGAGATAATCCAAAATGGATTAGTAAGG
CCATTCAGGATATGAAAAATAGGATCTTTGATCTCGTCGAAAATTCTTGTGACGGAGATACATTTCATAA
AGCATTGCAATGTTTGGTGGCTCTACGCAAAGGTTGCATCCTTGAGCAGGAACCAAAGCAGTTCAATGA
TTTCCTGTGCCACCTATCTAAATTTTGCCAAGAAAAAGACCTGAGAAGTTTCTGTCTATATCTCACATCTC
ATGAAATCACTTTGATAACCAAGGCAGAAGCTCCAGACAGTGAAATTTCAGAACATGAGGCTAGAAGCT
TTATGGTCAAGCCTGAACTTGACTCGCAAAATATGAAATCAGAGGCTAAAGCAGAGGATGATATTATGA
GTATATACCTGGGAGGGAAGggttcgccaaagaagaagcggaaggtctaa

Black color font: ku80DN; Purple font: SV40 NLS.

❖ **scfv-Ku80DN**

atgggccccgacatcgtgatgacccagagccccagcagcctgagcgccagcgtgggcgaccgcgtgaccatcacctgccgcagcagca
ccggcgccgtgaccaccagcaactacgccagctgggtgcaggagaagcccggcaagctgttcaagggcctgatcggcggcaccaacaa
ccgcgcccccggcgtgcccagccgcttcagcggcagcctgatcggcgacaaggccaccctgaccatcagcagcctgcagcccgaggact
tcgccacctacttctgcgccctgtggtacagcaaccactgggtgttcggccagggcaccaaggtggagctgaagcgcggcggcggcggc
agcggcggcggcggcagcggcggcggcggcagcagcggcggcggcagcgaggtgaagctgctggagagcggcggcggcctggtgca
gcccggcggcagcctgaagctgagctgcgccgtgagcggcttcagcctgaccgactacggcgtgaactgggtgcgccaggcccccggcc
gcggcctggagtggatcggcgtgatctggggcgacggcatcaccgactacaacagcgccctgaaggaccgcttcatcatcagcaagga
caacggcaagaacaccgtgtacctgcagatgagcaaggtgcgcagcgacgacaccgccctgtactactgcgtgaccggcctgttcgact
actggggccagggcaccctggtgaccgtgagcagctacccatacgatgttccagattacgctggtggaggcggaggttctgggggagga
ggtagtggcggtggtggttcaggaggcggcggaagcGGTTCGAAGCAACAAGACGCAGCAGATAAATTGGTTCAGA
TGTTGGATCTTGCACCACCTGGAAAACAGGAAGTGTTATCACCTGACTTCACACCTAATCCTGTTCTAGA
GCGTTACTACCGCTATCTTAACCTGAAGTCAAAGCACCCAGATGCAGCTGTTCCTCCACTTGATGAAACC
CTCAGAAAGATAACAGAACCTGATGTTGAACTTCTTTCTCAAAACAAGTCCATCATAGAGGAACTCCGTA
GGTCTTTTGAACTAAAAGATAATCCAAAGCTGAAAAAATCAGCAAGAAGAATAAAAGAaAGACCTTCAG
GATCAGATGAGGAGATAGAAGAATTCAACAAAGATGCTGATGTCAAAGCTATAGACTCCATGGAATACT
CAGCCAAAACAGAAGTTGAGAAAGTTGGAGATGTTAATCCTGTCAAAGACTTTGAGGATATGCTGTCTC
GAAGAGATAATCCAAAATGGATTAGTAAGGCCATTCAGGATATGAAAAATAGGATCTTTGATCTCGTCG
AAAATTCTTGTGACGGAGATACATTTCATAAAGCATTGCAATGTTTGGTGGCTCTACGCAAAGGTTGCAT
CCTTGAGCAGGAACCAAAGCAGTTCAATGATTTCCTGTGCCACCTATCTAAATTTTGCCAAGAAAAAGAC
CTGAGAAGTTTCTGTCTATATCTCACATCTCATGAAATCACTTTGATAACCAAGGCAGAAGCTCCAGACA
GTGAAATTTCAGAACATGAGGCTAGAAGCTTTATGGTCAAGCCTGAACTTGACTCGCAAAATATGAAAT
CAGAGGCTAAAGCAGAGGATGATATTATGAGTATATACCTGGGAGGGAAGggttcgccaaagaagaagcgga
aggtctaa

Orange color font: scfv sequence; red color font: HA tag; green color font: linker; black color font: ku80DN; Purple font: SV40 NLS.

❖ ***SlBRCA1* sequence, cloned using tomato total cDNA**

ATGGCAGATATTTCGCACCTTGAAAGAATGGGAAGAGAGCTCAAATGCCCCATTTGCTTGAGTCTGTTC
AATTCTGCTGTTTCACTTACATGTAATCATGTATTTTGCAATTTATGTATTCAAACTGGTATGAAATCTGG
ATCCAATTGTCCGGTGTGCAAAGTTCCATTTCATCGCAGAGAAATCCGACCTGCTCTCCACATGGATAAC
TTGGTGAGCATCTATAAGAACATGGAAATTGCTTCAGGAGTCAATATGTTTCTCACTCAATCCAATCCTTC
CACAAAATTACCAGGGGAAAACACTCGATCTAATGGCGAAAAAGTTTGTGGATTCCAAGAGACACCTAA
AACTGTAACAGAAGCTCCAGCGACAGACAATCAGAAAAGAAAAAGAGGGAAAGGATCAAAAAGATCTT
CAGGGTGCAACAAAAAAATTTCCGGATCAAATCTTATTAGACCTTCTTTTCCAACAAAGAAGAGAGTACA
GGTGCCACAATATCCACCTTCAGAGACTCCACCACCTACAAAGTTAGTTGATGGAAATGGCAAATCCATC
ACCGATGAAGTTCAGAAACCATTGGTAATTGAGAGAGATAGGTCTATGCTAAATGAAAAAGGAGAACC
TGTGCTATCTCCATTCTTCTGGCTGAGAGAAGAGGATGTAGATAAATCAAGTCAGCAAACAGATGGGGA
CGTTATCATGGATACTCCTCCAGCTTTTCCATCCTTCAGCGATATGAAAGACTTGGATGATGAGGTTCACT
GCGAAATGACTCCAAAAAGTGGACCCTATGATGCAGCAAATGGAGCAGATCTTTTTGACAGTGAGATGT
TTGACTGGACACAAAGAGCTTGCTCCCCTGAACTTTGTTCTAGTCCCTTTAAGATGAAGCTTAAGGATAC
TATTGATTCTGCTGAAGCTCAGGAAAAGACTCAAGCTCACTCAGTTGAGGAAAGTGACATTAATGCATC
AGCAACTGAAAACAGAACAGCTGTGGAAAATGAAAAGGGTACTGATAAAGGACAGCTGAGTTCACCCG
CGATATTTTCTCCTGTAAATAAAACCACTAGTAGAAAAGACGTTGTTTGCAAGTCTAGCAGGAGCAAGTC
ATTGAGAAGTAGCCAGAAGAAACAAGGAAAAAACATAATTGGAGAATTATCAGAAGTTCATGATGCTTC
ACTAAAAGCAGCTGAAAACACTATGAAGAACAATCAGGATAATGCCAATGCATTTATCTCGAATAAGAA
GGATTCGAAAAACAAGAAAAAGGGTAGATCCTCTAGAAATGTCACTGAGTCAGTTGTAGAAGACATTTC
CACTTCATGTGGTGCTAAAAGACTTCGCAAGGGCAATAACTCAAAGTCCTTCAACTTGTCTACTATTGTG
AATCAGGAAAAACATAGTGAAGGAAGTGTCGAGACACTTGACTTGAAGACGCATAATATATTCCGCAAG
GGATCATTACGTGAACAAGCTAAAAACTGTTTTGGACCAAAAGGCGGAAAGAAAACTGCTGTGTGTAAC
ATTCCACAAATTCAAGATGAAGCTTTGCCTTTCGAATCAGCAAATAGATTGATTCCCATGGATAACAAGA
AACCTACTCGTAGTACAAAACTGAAGAAATGTGAGCTTGGTAGTGACAACAAGCTTCATGGGAAGAAAA
AAGTGAAGTTTTCTGATGATGGGCAGCTTGCTGACAAGGATAATATTACGCTTCAAAAGATTCAGAAGA
GAGTGCTCAATTCATTGGAAACAGATAAATCTGTTTTGAACTCGAATGATTCAGTTTTGCAGAAGTGTGA
GGCAAGCCAAAGCAAAATACAATGTGCTTTCTGCCGTTCAGCAGAGATATCTGAGGTTTCAGGAATCAT
GGTAAGCTACCTTAATGGAAACCCTGTCAAAGAAGATGTCAATGGAGCACCAGGTGTTATACATGTTCA
CAAATATTGTGCAGAATGGGCCCCTAATGTATATTTTGGAGACGATGATGTGGTCAACCTTGAATCTGAA
CTGAAGAGGAGTCGAAGGATCACTTGTTTTTTCTGTGGAGTAAAGGGGGCAGCTCTTGGGTGTTATGAG
ATGAGTTGTCGCAAAAGCTTTCATGTTCCTTGTGCCAAATTGACACCAGAGTGTAGATGGGATTCTGATA
ACTTTGTTATGTTATGCCCTTTGCATGCTAACTCTAAGTTGCCATCCGAGATCCCAGGAAAACAGACAAA
GATTGGAGATAGCATTAAAAGAAATTCTCGTATCCACCAACCCAATGTCTCAGCAACACCTGATAATGGT
GCTACTTTGCAGTGGAAGTCTCAGAAGAAGAATAAGAACTTAGTGCTCTGTTGTTCAGCTCTAACCGCA
GATGAAAAAGAGCTTGTTTCTAAATTAAAGAGGTTGTCTGGCGTGACAGTAGTCAAGAACTGGGACCTA
AGTGTCACCCATGTCATTGCTTCTACTGATGAGAAAGGAGCATGCCGAAGAACTCTCAAGTATTTGATG

GGTGTCTTGGCTGGGAAATGGATAATGAGCATCAACTGGATTATTGCTAGCTTGGAAGCCACAGAATAT GTCGATGAACAACAATACGAGATTAAAATAGATACTCATGGCATTGTGGATGGGCCTAAGCTTGGAAGA TTGAGGATTTTAAACAAGCAACCAAAGCTTTTCAATGGATACAAGTTCTTTTTTATGGGCGACTTTTTATC TTCATACAAGAGCTACCTACATGACCTTGTTATTGCTGCTGGAGGAATTGTCCTTAACAGGAAGCCTATT GCACTGGATCAGGAAATTCTTTCACCCGGATGCCCTCTACTGTTTGTAATTTATAGTCATGAACAACTTGA TCAGTGCGAAGGAAGTGAAAAAATTTCAATTATAGCGCGCAGAAGATCTAATGCAGAAGTTTTGGCTAG TTCAACTGGAGCTGTAGCTGCCAGCCACTCCTGGATTCTAAACTGCATTGCTGGCTCTAGGTTGTTGGAG CTGGAATAG

❖ **Linker sequence for N-terminal fusion**

GGAATGATGGCCCCCAAGAAGAAGCGCAAGGTGGGACGCGTCTGCAGGATATCAAGCTTGCGGTACCG CGGGCCCGGGATCGCCACC

Red font: SV40 NLS sequence

❖ **Linker sequence for C-terminal fusion**

AGCCTGGGCAGCGGCTCCCCCAAGAAAAAACGCAAGGTGGAAGATCCTAAGAAAAAGCGGAAAGTGG ACGGCATTGGTAGTGGGAGCGGAGGTGGGGGATCTGGTTCG

Red font: SV40 NLS sequence

❖ **crRNA expression cassettes and donor sequences for *SlHKT1;2* locus**

- U6-crR1-2.20[HKT1;2] expression cassette:

TGATCAAAAGTCCCACATCGATCAGGTGATATATAGCAGCTTAGTTTATATAATGATAGAGTCGACA TAGCGATTGtAATTTCTACTAAGTGTAGATACTATTCACCACAGTATCAATAATTTCTACTAAGTGTAGA TCCTACAAATGAAAACATGATTTTTTT

Red font: AtU6 promoter; green font: LbCas12a scaffolds; light blue font: LbCas12a_gRNA1, 20nt; orange font: LbCas12a_gRNA2, 20nt; black G: transcription start; black TTTTTT: termination sequence.

❖ **crRNA expression cassettes and donor sequences for *SlEPSPS1* locus**

- Dual U6-crR1-2.23[EPSPS1] expression cassette:

TGATCAAAAGTCCCACATCGATCAGGTGATATATAGCAGCTTAGTTTATATAATGATAGAGTCGACA TAGCGATTGtAATTTCTACTAAGTGTAGATGCGAAGTCCTCTACTGTCTCTAATTTCTACTAAGTG TAGATCATATGGAGGAGGTCTGTGTTTTTTT

Red font: AtU6 promoter; green font: LbCas12a scaffolds; blue font: LbCas12a_gRNA1, 23nt; purple font: LbCas12a_gRNA2, 23nt; black G: transcription start; black TTTTTT: termination sequence.

❖ ***SlCAB13* donor sequence**

TGGAAAATGACTTTGATCGTATCAAGCCAATGAATAAAAAAAACTTTATAAATTCGATAATCTCCGAATATATC
CCGTGAATAACATATATTACTTGCGTTTCTCTTCATTATCTTTTTTTAAAAATATCTGTATATAGATATCTTCAGC
TAATTCTTTTAGTGAAATATGTTTATAGAGCAATGCACTTGTAATTATATATGGTCGTAAAAGTATGGTTTAAT
TCAACTCTTGGCTTGATTAAGCACAGAGGCATGAAGGGGACTCCTTGAGCACATAATTAATTAGTTGCATGGG
GTGCCATGAATTTGTTTCTTCAATTCAATTACTTTTTCAATTTCAATAACTCGTGGTTATAGAATAGAGTGTGAA
CAACATATACAAGACACGAGTTATAGAATAGAGTGTGAACAACATATACAAGATTGGAAATTGACAATTAAA
ATTCATTTAGTATAGCCACTAGAAAAAGTTGGCTAATCATACGCAGTAGACTCGAATTACTTTGGTGCGCATG
AGTCTAAACATTTTGGCAAGTTGGGAAACATGAAACAACGCTTACAATAAAGTGCATCGATATAAAAGTATGT
TTAAATACCATAAAATTTCATATTTAAATTTTAGCCTGACAAAATAGTAATGAGCGATTTATTCTCATCTATCAA
AATCTTAATAAATAAAGTTATTTAATTTTTATATTAATTACAAATTGATTCGTACGCCACAATAATAAAATAAAA
TCCAAAAAGCTTGACCAATGACCATATATTACATTGTGTACTACTATATGTTAGCAAGATAAAACTCTGGATAG
AAGGATATGACATGAAGATACTCAAAATCTACTCAACACTTCTCATTGGTTGATACAAAAATTTCAAAACTATT
TCACACCACAATTTTATAAAACACAAGAACCAGAATTGTTAAATGGGGCCCCTCCAGATATTTTTCCAACAGTT
ATTCTTTATATATAAGTATATAAATTGTGCAAGAAACTTAGTGAAAATTGTGAGGTCCAATTAAcTAgAAGAGA
AATTAAGCAATAGAGAAAATGGCAGCAACAGGTAGCTCAGCCACAGTTGTTAGAGCAACTCCATTTTTGGGC
CAaACCAAgTATGCTAACCCCCTAAGGGATATAGTTCCTATGGGCTCTGCCAGATTCACCATGGTAACTTTTTA
TTTTTCTTTTAAGTGTTTTTATAATTGAGTTCGATATTTAATATTATTGAACTTCAAATAAATTTATGATTTGTGT
AAAAAGATACTGAACCTGTTATGTTTTATCAGAGTAATGATTTGTGGTATGGACCTGACCGTGTCAAGTACTT
GGGACCATTTTCTGCTCAAACTCCTTCATACTTGACTGGAGAATTCCCTGGTGATTACGGATGGGATACTGCTG
GTTTATCTGCTGATCCCGAGGCCTTTGCTAAGAACAGAGCTCTTGAGGTACTTTCACTTTTTCTTTTACTAGTAG
TAAGTTTGTTTAAAATAAGTAACAGACATTTGCGTGACTAAAATTCATTTTTAGCTAAAAAAGTTTGAAAAATA
AAAAAATTATCTTTTTCGTTTTATTTTATGTGACACTATTTGACTTGACACAGTGTTTTAAGAAGAAGAAAAAAT
GGTCTAAACCATTTCTTAGATATTGTGTAGTTGAAAATCATCTCATTAAGGATAAAAGAGGTATTTTAAAGCTA
ACTTGTTTCTAATTATAGTGATATTTTTTTTTAACGGATAAAAATGAAAGAATATCACATAAAATAGGACAGAG
GAATATCATTTTTGTTGGGACAAAACAAAAAAGTGAAGCACGCCATAGAAAATGTACAGATTTGAGACTCGT
AACGATTAATTTAGTATTAAGACATAGTTGATGGATTGATATGAATTGAAATTGTGTTAATTATTCTAGGTTAT
CCATGGGAGATGGGCCATGCTTGGAGCTTTTGGTTGCATTACACCAGAAGTTCTTGAAAAATGGGTAAAAGT
GGACTTCAAAGAACCAGTATGGTTCAAAGCTGGAGCCCAGATCTTCAGTGAAGGTGGGCTGGACTATTTGGG
CAACCCAAACCTTGTCCATGCTCAGAGC

Blue font: LbCas12a_gR1.CAB13 binding site; Purple font: LbCas12a_gR2.CAB13 binding site; yellow highlighted font: modified PAM and LbCpf1 gRNA core region to prevent recut after HDR editing; red font: AAATTGTGA inserted sequence.

- **crRNA expression cassettes and donor sequences for *SlCAB13* locus**
  - Dual U6-crR1-2.20^{CAB13} expression cassette:

TGATCAAAAGTCCCACATCGATCAGGTGATATATAGCAGCTTAGTTTATATAATGATAGAGTCGACA
TAGCGATTGtAATTTCTACTAAGTGTAGATATTAATTGGACCTCACTAAGTAATTTCTACTAAGTG
TAGATGTCTGGCCCAAAAATGGAGTTTTTTT

Red font: AtU6 promoter; green font: LbCas12a scaffolds; blue font: LbCas12a_gR1.CAB13, 20nt; purple font: LbCas12a_gR2.CAB13, 20nt; black G: transcription start; black TTTTTT: termination sequence.
