# Supplementary material for: Enhancing CRISPR-Cas-based gene targeting in tomato using a dominant-negative ku80
Source: Hortic Res. 2024 Oct 23;12(2):uhae294. doi: 10.1093/hr/uhae294 (PMC11789525; doi:10.1093/hr/uhae294)
Supplement: Web_Material_uhae294 [file web_material_uhae294.zip › 24.06.08_Supplemental Tables.pdf]

Supplementary Table 1. The impacts of overexpression of free KUDN on microhomology length and frequency

No.	Targeted locus	Construct	Total read	Indel frequency (%)	MMEJ read	MMEJ frequency (%)	MMEJ/indel ratio (%)	Total reads					Frequency (%)					Normalized MMEJ/indel ratio (%)
								2-bp MH	3-bp MH	4-bp MH	5-bp MH	6-bp MH	2-bp MH	3-bp MH	4-bp MH	5-bp MH	6-bp MH	
1	*SlHKT1;2*	ttLbCas12a	194596	26.34	7495	3.85	14.62	4007	953	1204	453	410	1.97	0.47	0.59	0.22	0.20	1.00
2		ttLbCas12a, -gRNA	190331	0.06	12	0.01	10.04	7	1	1	3	0	0.00	0.00	0.00	0.00	0.00	0.69
3		ttLbCas12a, free KUDN	195458	35.63	16341	8.36	23.46	5294	2073	1915	6615	126	2.52	0.99	0.91	3.15	0.06	1.60
4	*SlEPSPS1*	ttLbCas12a	141624	25.75	5169	3.65	14.18	4716	328	0	0	0	2.79	0.19	0.00	0.00	0.00	1.00
5		ttLbCas12a, -gRNA	188699	0.07	6	0.00	4.69	4	2	0	125	0	0.00	0.00	0.00	0.07	0.00	0.33
6		ttLbCas12a, free KUDN	168199	23.71	6672	3.97	16.73	5840	561	168	0	0	3.60	0.35	0.10	0.00	0.00	1.18

Supplementary Table 2. The impacts of the recruitment of KUDN to the targeted sites by the Suntag system on GT and indel efficiency

No.	Targeted locus	Construct	Rep 1			Rep 2			Rep3			Average			SEM	
			Total reads	GT efficiency (%)	Indel rate gRNA1 (%)	Total reads	GT efficiency (%)	Indel rate gRNA1 (%)	Total reads	GT efficiency (%)	Indel rate gRNA1 (%)	Total reads	GT efficiency (%)	Indel rate gRNA1 (%)	GT efficiency (%)	Indel rate gRNA1 (%)
1	*SlHKT1;2*	ttLbCas12a	41385	0.031	24.678	97080	0.10	45.71	121976	0.058	50.353	260441	0.063	40.247	0.020	7.899
3		ttLbCas12a + 10xsuntag-KUDN	96018	0.024	3.799	87546	0.06	14.80	109884	0.015	11.565	293448	0.031	10.055	0.013	3.264
4	*SlEPSPS1*	ttLbCas12a	55518	0.022	6.976	42647	0.10	41.22	134686	0.014	20.392	232851	0.045	22.862	0.027	9.961
6		ttLbCas12a + 10xsuntag-KUDN	83297	0.012	2.295	60183	0.03	6.65	77297	0.021	6.197	220777	0.020	5.048	0.005	1.383

Supplementary Table 3. The targeted loci and gRNAs used in the study

No.	**Locus**	**Accession**	**Primer name**	**Sequence (5' - 3')**
1	*SlHKT1;2*	Solyc07g014680	LbCpf1_gR1.HKT12	ACTATTCACCACAGTATCAACTT
2			LbCpf1_ gR2.HKT12	CCTACAAATGAAAACATGATGAT
3	*SlEPSPS1*	Solyc01g091190	LbCpf1_ gR1.EPSPS1	CAAGGAATAGTTGGATTTCTTCC
4			LbCpf1_ gR2.EPSPS1	AATCGTTCCTTCTTCGTGCCATT
5	*SlCAB13*	Solyc07g063600	LbCas12a_gR1.CAB13	ATTAATTGGACCTCACTAAG
6			LbCas12a_gR2.CAB13	GTCTGGCCCAAAAATGGAGT

Supplementary Table 4. Primer sequence used in this study.

No.	Loci	Primer name	Sequence (5' - 3')	Note
1	*SlHKT1;2*	UPHKT12-F1	TTCACATGCTTTGACCCATAAA	1st PCR
2		DNHKT12-R1	CTCTTCCTATAAACGTGCACTCA	
3		nHKT12-F2	ACACTCTTTCCCTACACGACGCTCTTCCG ATCTCCCTAGCGCCAAACAAATC	2nd PCR
4		nHKT12-R2	GTGACTGGAGTTCAGACGTGTGCTCTTCC GATCTGGGATAAGAATGAGAAGAAGACCT GAATT	
5		HKT12-sF1	CAAAGATTATGAGCTAGGGAATGT	Sequencing primer
6	*SlEPSPS1*	UPEPSPS1-F2	ACATGTAAGTTAGACAAGAGCTAGG	1st PCR
7		DNEPSPS1-R1	GGGAGTGAGTGCATACTTGTT	
8		EPPE-F2	ACACTCTTTCCCTACACGACGCTCTTCCG ATCTGGCAGTTTCCTGTCGGTAA	2nd PCR
9		nEPSPS1-R2	GTGACTGGAGTTCAGACGTGTGCTCTTCC GATCTCAACACTGGAAAAAAAGAAGAAAAA A	
10		EPSPS1-sF1.2	TGGTGTCCTGAACAACTCATAC	Sanger sequencing
11	*SlCAB13*	UPCAB13-F1	ATCGAGAGTCCTTACTCTTCACG	1st PCR
12		DNCAB13-R1	CTCCAGGAAGCCCATTAATTCT	
13		CAB13-F2	ACACTCTTTCCCTACACGACGCTCTTCCG ATCTCAGTTATTCTTTATATATAAGTATATA A	2nd PCR
14		CAB13-R2	GTGACTGGAGTTCAGACGTGTGCTCTTCC GATCCTATATCCCTTAGGGGGTTAGC	
15		SlCAB13-sF2	GTGTGAACAACATATACAAGATTGG	Sanger sequencing
16		SlOR-sF2	GACGCATCTTCATACGCTACTT	Sanger sequencing
17	GT tool	RB-qF2	CTCTTAGGTTTACCCGCCAATA	T-DNA
18		GR-F1	TTGAGATGAGCACTTGGGATAG	
19		GR-F1	TTGAGATGAGCACTTGGGATAG	Replicon
20		pNOS-cR1	AACGTGACTCCCTTAATTCTCC	
21	*SlGAPDH*	GAPDH-F1	CCATAACCTAATTTCTCTCTC	Internal control
22		GAPDH-R1	GTCATGAGACCCTCAACAAT	

Supplementary Table 5. Primers used for off-target analysis

No.	Potential off-target site	Chromosome location	Primer	Sequence (5'-3')	Length (nt)	Note
1	EPSPS1-OT1	SL4.0ch05-60580370..60584370	EPSPS1-OT1-F1	CCTACTCAGTGGGAGCTTAGT	21	PCR
2			EPSPS1-OT1-R1	AATGCCCGCCCATAAAGAA	19	
3			EPSPS1-OT1-sF1	TTCTTATGGTTGCACTTCTCA	21	Sequencing
4	CAB13-OT1	SL4.0ch01-56416253..56420253	CAB13-OT1-F1	ATTTGGTGCCCTGGTCTTAG	20	PCR
5			CAB13-OT1-R1	ATCCCGACCATCTTCGTATTG	21	
6			CAB13-OT1-sF1	ACCTGCAGGCCTTTGATTT	19	Sequencing
7	CAB13-OT2	SL4.0ch12-4304011..4308011	CAB13-OT2-F1	GATAGCCATCTCATTAATTTCAAGACC	27	PCR
8			CAB13-OT2-R1	TCCACCGTCACTGAAGATTTG	21	
9			CAB13-OT2-sF1	CTCCACTTTAGGCCTCGATTAT	22	Sequencing
