# Supplementary material for: Enhancing CRISPR-Cas-based gene targeting in tomato using a dominant-negative ku80
Source: Hortic Res. 2024 Oct 23;12(2):uhae294. doi: 10.1093/hr/uhae294 (PMC11789525; doi:10.1093/hr/uhae294)
Supplement: Web_Material_uhae294 [file web_material_uhae294.zip › 24.09.07_Supplemental Figures kjy1.pdf]

Supplemental Figures

Enhancing CRISPR-Cas-based gene targeting in tomato using a dominant-negative *ku80*

Tien Van Vu[1, §,*], Ngan Thi Nguyen[1, §], Jihae Kim[1, §], Minh Huy Vu[1], Young Jong Song[1], Mil Thi Tran[1,4], Yeon Woo Sung[1], Jae-Yean Kim[1,2,3,*]

[1]Division of Applied Life Science (BK21 Four Program), Plant Molecular Biology and Biotechnology Research Center, Gyeongsang National University, Jinju 660-701, Republic of Korea.

[2]Division of Life Science, Gyeongsang National University, 501 Jinju-daero, Jinju 52828, Republic of Korea.

[3]Nulla Bio Inc 501 Jinju-daero, Jinju 52828, Korea.

[4]Current affiliation: Biological Resource Center, Korea Research Institute of Bioscience and Biotechnology (KRIBB), Jeongeup, 56212, Republic of Korea.

[*]Correspondence: Tien Van Vu: tienvu.agi@gmail.com (ORCID: 0000-0002-6369-7664); Jae-Yean Kim: +82-(0)55-772-1361, kimjy@gnu.ac.kr (ORCID: 0000-0002-1180-6232).

[§]These authors contributed equally to this study.

Running title: Enhanced CRISPR-Cas-based GT in tomato.

```
Range 1: 4 to 704 GenPept  Graphics                                 ▼ Next Match  ▲ Pre

Score           Expect  Method                          Identities      Positives       Gaps
206 bits(524)   8e-57   Compositional matrix adjust.    205/761(27%)    357/761(46%)    95/761(12%)

Query  6     NKAAVVLCMDVGFTMSNSIPGIESPFEQAKKVITMFVQRQVFAENKDEIALVLFGTDGTD  65
             NK A+VL +DVG +M + +P IE       KV ++ +Q+++     DE+  VLFGT  T
Sbjct  4     NKEALVLVIDVGPSMHSVLPEIE-------KVCSLLIQKKLVFSRYDEVGFVLFGTADTK  56

Query  66    NPLS---GGDQYQNITVHRHLMLPDFDLLEDIESKIQPGSQQADFLDALIVSMDVIQHET  122
             N L    GG  Y+++TV R++ + D DL++ ++ K+  GS   DFLDA++V  D++
Sbjct  57    NELKEEIGG--YEHVTVLRNIKVVDEDLVDALQ-KLPRGSVPGDFLDAIVVGTDMLIKFG  113

Query  123   IGKKFEKRHIEIFTDLSSRFSKSQLDIIIHSLKKCDISLQFFLPFSLGKEDGSGDRGDGP  182
                K  KR + + T+  SR                        PF   KED
Sbjct  114   RTNK-AKRRLCLITNAVSRIKD---------------------PFEGTKEDQVNTIAT--  149

Query  183   FRLGGHGPSFPLKGITEQQKEGLEIVKMVMISLEGEDGLDEIYSFSESLRKLCV------  236
              ++   G    +  +  + K+  E  + +M   E  D L  ++S   S + + V
Sbjct  150   -QMTAQG--IKMDCVIVRMKQDRETNRSIM---EENDFLMSVFSNKSSSKVVYVESPTSL  203

Query  237   FKKIERHSIH----WPCRLTIGSNLSIRIAAYKSILQERVK--KTWTVVDAKTLK--KED  288
                +   +I     +   L I + L I++  YK   +E+    K ++     T K    D
Sbjct  204   LGALRTRNISPVTIYRGDLEISAQLKIKVWVYKKTSEEKFPTLKKYSERTPTTDKFGAHD  263

Query  289   IQKETVYCLNDDDETEVLKEDIIQGFRYGSDIVPFSKVDEEQMKYKSEGKCFSVLGFCKS  348
             I+ E    + +D    V  E  I+GF+YG  +VP S  + E +K+K E K   +LGF  S
Sbjct  264   IKVEYENKIIEDPNKVVPPEQRIKGFQYGPQVVPISSAELEAVKFKPE-KSVKLLGFTDS  322

Query  349   SQVQRRFFMGNQVLKVFAAR-DDEAAAVALSSLIHALDDLDMVAIVRYAYDK-RANPQVG  406
             S + R +++ +  + +F A   ++ A +ALS+L  A+ +++ VAIVR  + + + N  VG
Sbjct  323   SNIMRHYYLKD--VNIFIAEPGNKNAILALSALARAMKEMNKVAIVRCVWRQGQGNVVVG  380

Query  407   VAFPHI--KHNYECLVYVQ-LPFMEDLRQYMFSSLKN-SKKYAPTEAQLNAVDALIDSMS  462
             V  P++  K N     Y   LPF ED+R + F S  N      P E Q +A D L+  +
Sbjct  381   VLTPNVSDKDNTPDSFYFNILPFAEDVRDFQFPSFSNLPSSMQPNEKQQDAADKLVQMLD  440

Query  463   LAKKDEKTDTLEDLFPTTKIPNPRFQRLFQCLLHRALHPREPLPPIQQHIWNMLNPPAEV  522
             LA    K + L   F     PNP  +R ++ L  ++ HP   +PP+ + +  +  P  E+
Sbjct  441   LAPPG-KQEVLSPDF----TPNPVLERYYRYLNLKSKHPDAAVPPLDETLRKITEPDVEL  495

Query  523   TTKSQIPLSKIKTLFPLIEAKKKDQVTAQEIFQDNHEDGPTAKKLKTEQGGAHFSVSSL-  581
              ++++  + +++  F L +  K  + +A+ I     ++ P+    + E+      V ++
Sbjct  496   LSQNKSIIEELRRSFELKDNPKLKK-SARRI-----KERPSGSDEEIEEFNKDADVKAID  549

Query  582   -----AEGSVTSVGSVNPAENFRVLVKQK------KASFEEASNQLINHIEQFLDTNETP  630
                  A+  V  VG VNP ++F  ++ ++        + ++  N++ + +E   D +
Sbjct  550   SMEYSAKTEVEKVGDVNPVKDFEDMLSRRDNPKWISKAIQDMKNRIFDLVENSCDGDT--  607

Query  631   YFMKSIDCIRAFREEAIKFSEEQRFNNFLKALQEKVEIKQLNHFWEIVVQDGITLITKEE  690
              F K++ C+ A R+  I   E ++FN+FL  L +  + K L  F   +    ITLITK E
Sbjct  608   -FHKALQCLVALRKGCILEQEPKQFNDFLCHLSKFCQEKDLRSFCLYLTSHEITLITKAE  666

Query  691   ASGSSVTAEEAKKFLAPKDKPSGDTAAVFEEGGDVDDLLDM  731
             A  S ++  EA+ F+    KP  D+  +  E    DD++ +
Sbjct  667   APDSEISEHEARSFMV---KPELDSQNMKSEAKAEDDIMSI  704

Query = hamster KU80; Sbjct = tomato KU80
```

Figure S1. Protein alignment with NCBI blast between the hamster Ku80 and tomato Ku80 peptides. The red discontinuous box denotes the starting or KUDN peptide.

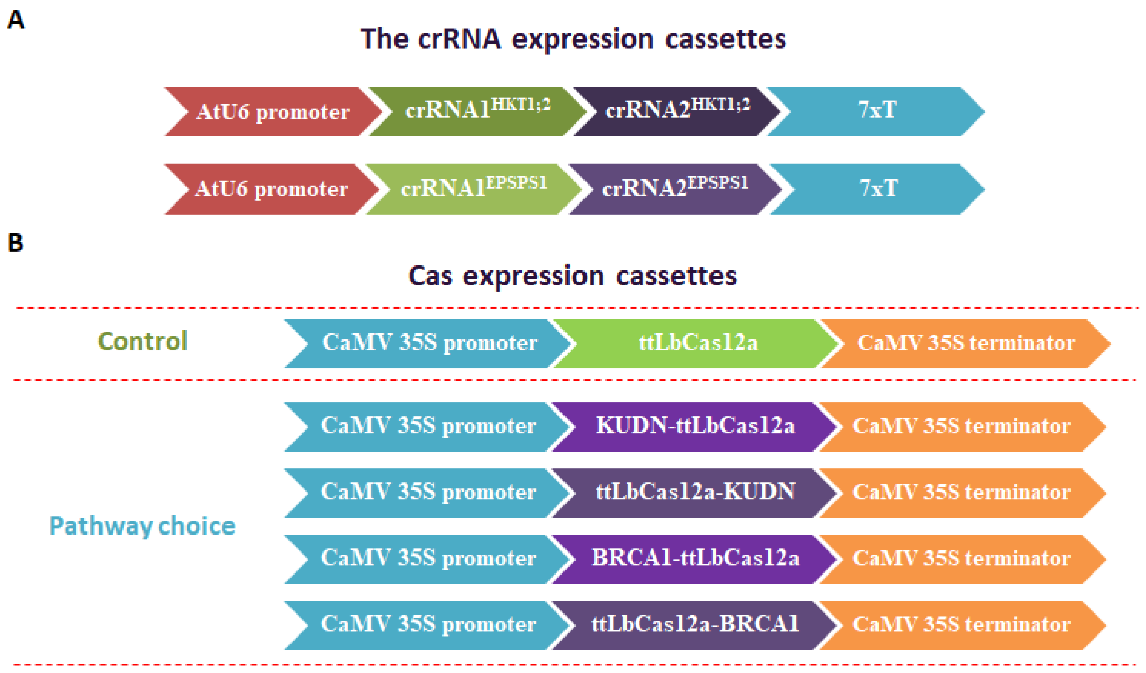

Figure S2. The expression cassettes of gRNAs and Cas protein used in the study. **A** Two crRNAs (LbCas12a scaffold and gRNA) were cloned in tandem repeats under the driving of the AtU6 promoter and terminated by seven T (7xT) for cleaving each targeted site. **B** The Cas protein expression cassettes were designed for the Control, End resection facilitation, Pathway choice, and Cell cycle synchronization using the CaMV 35S promoter with or without the TRP1 intron 1 and the CaMV 35S terminator.

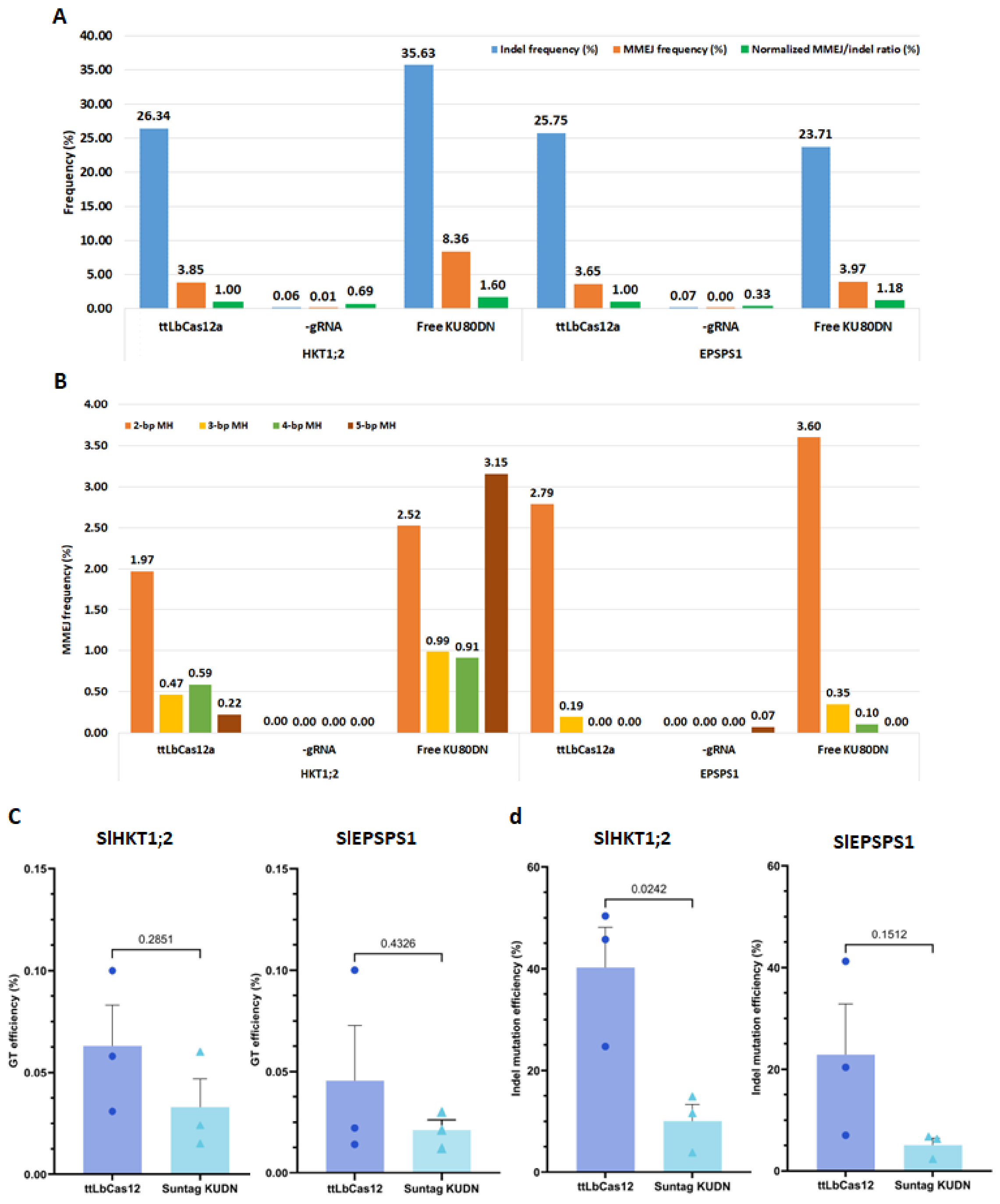

Figure S3. The impacts of the overexpression of free and Suntag-recruited KUDN on the editing efficiency in tomato at the callus stage. A-B. The impacts of free KUDN on the editing efficiency (**A**) and the MMEJ efficiency with different microhomology lengths (**B**). **C-D**. The impacts of Suntag-recruited KUDN to the targeted sites on the GT (**C**) and indel mutation (**D**) efficiency at the callus stage were assessed by targeted deep sequencing. P-values of the t-test for pair-wise comparisons are indicated on the top of the bars. All data points are shown as dots in the plots in (**C-D**).

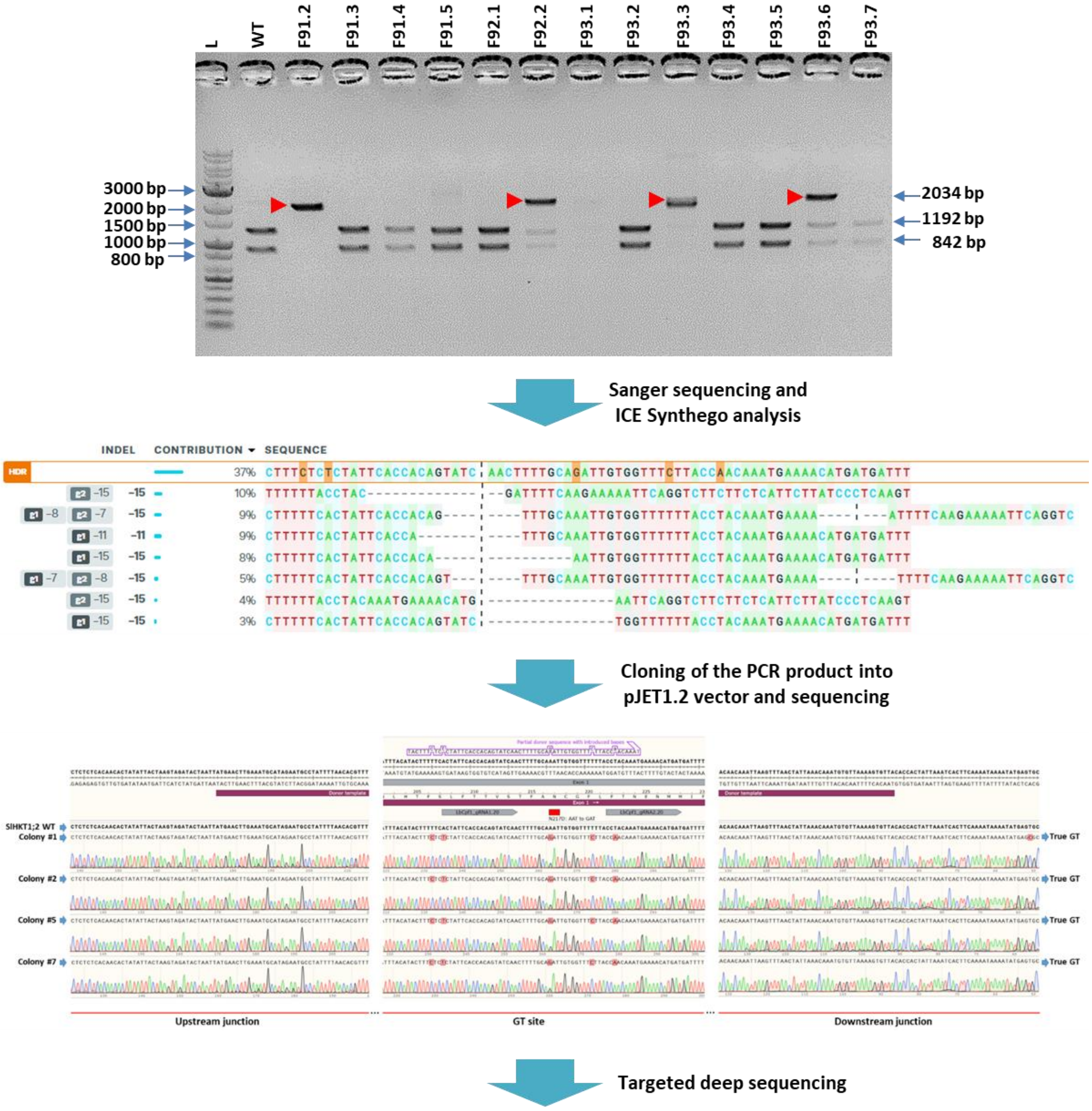

Total reads	GT frequency (%)	Indel frequency (%)
6058	50.330	48.151

Figure S4. The step-by-step procedure to confirm a GT0 event. The CAPS assay might first screen transformants. Alternatively, the PCR products amplified from the targeted sites of GT transformants were directly sequenced and analyzed by ICE Synthego. Potential GT events were then validated by (1) cloning the PCR products into pJET1.2 plasmid and sequencing and (2) targeted deep sequencing.

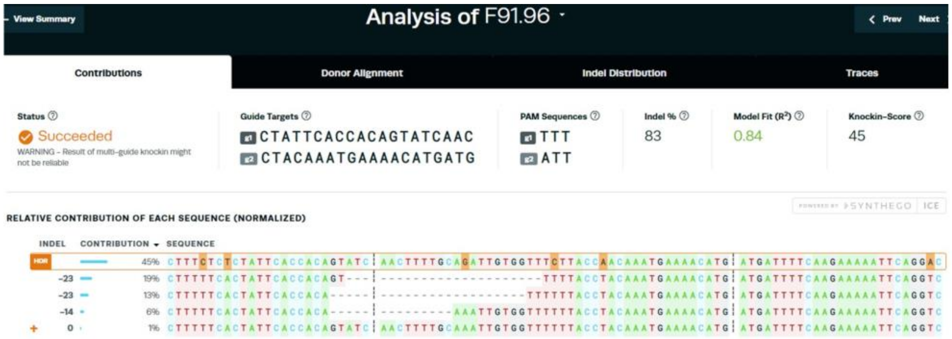

ICE Synthego analysis found the GT allele from the plant

- **pJET1.2 cloning and sequencing**

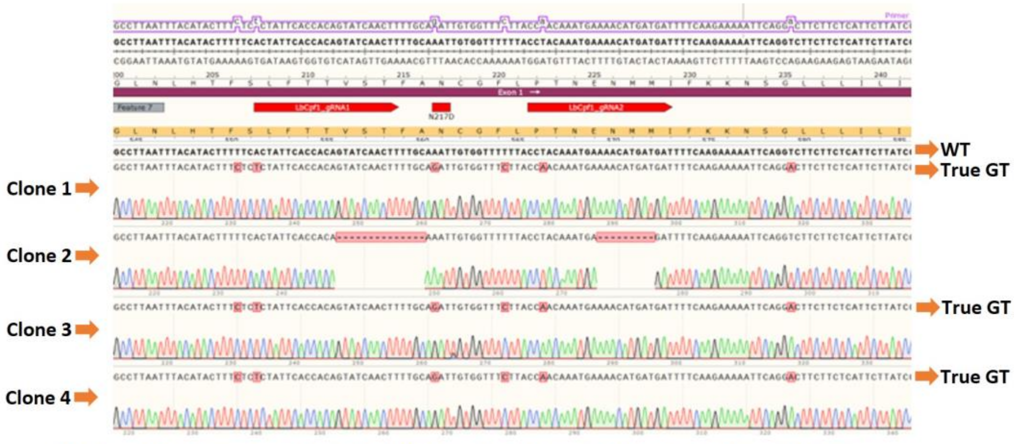

3/4 clones appear to carry perfect GT alleles. Junction sequencing is conducting for validating it.

- **Miniseq data**

Total Sequences	With both indicator sequences	More than minimum frequency	Insertions	Deletions	Indel frequency	HDR frequency
124307	54646	54646	0	28754	28754 (52.6%)	25029 (45.8%)

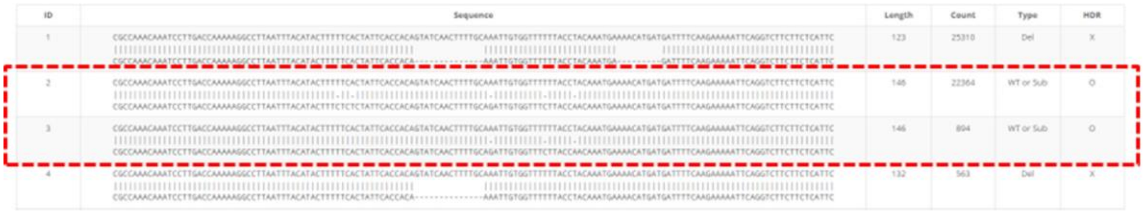

Figure S5. Identification and validation of the *SlHKT1;2* GT0 event, F91.96. Top panel: ICE-Synthego analysis identified F91.96 as a potential GT event confirmed by pJET1.2 cloning and sequencing of PCR product and targeted NGS. Sequencing data revealed that 3/4 of colonies contained desired base changes at the GT site and no additional change at the upstream and downstream junctions.

A

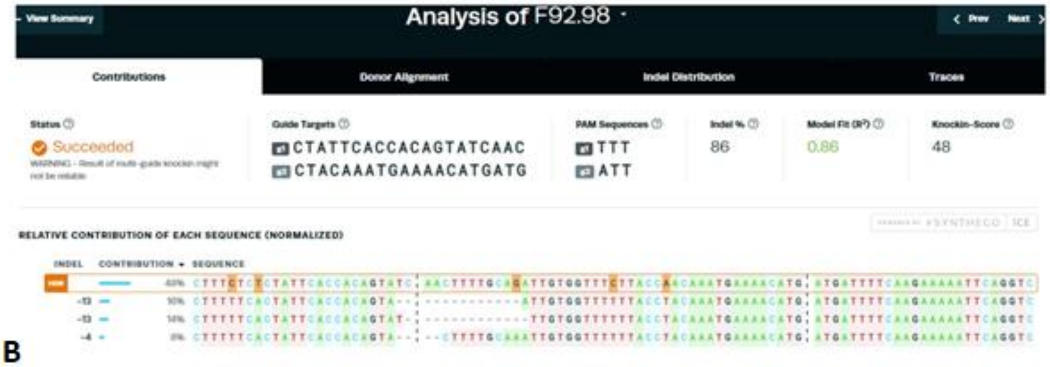

B

Total reads	GT efficiency (%)	Indel rate gRNA1 (%)
6058	50.330	48.151

C

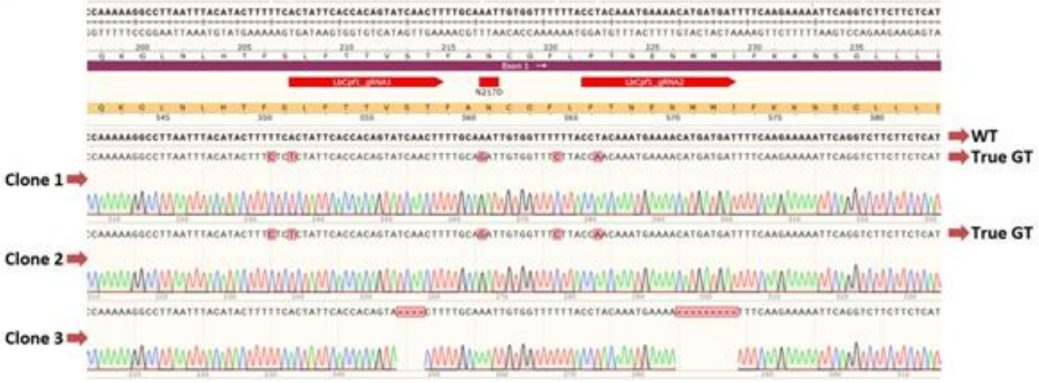

Figure S6. Identification and validation of the *SlHKT1;2* GT0 event, F92.98, obtained by the NKUDN-GT construct. A ICE-Synthego analysis identified F92.98 as a potential GT event confirmed by targeted NGS (**B**) and pJET1.2 cloning and sequencing of PCR product (**C**). Sequencing data revealed that 2/3 of colonies contained desired base changes at the GT site and no additional charge at the upstream and downstream junctions.

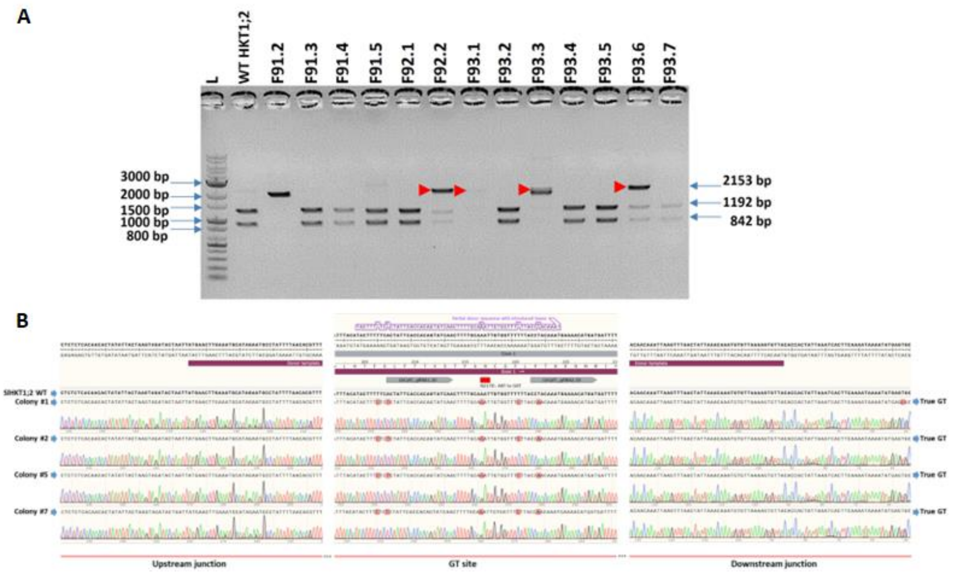

Figure S7. Identification and validation of the *SlHKT1;2* GT0 event, F93.6, obtained by the CKUDN-GT construct. A CAPS assay identified F93.6 as a potential GT event. The potential GT bands are denoted with red rectangles. **B** Cloning the F93.6 PCR product into pJET1.2 and sequencing revealed a true GT allele. Sequencing data revealed that 3/7 colonies contained desired base changes at the GT site and no additional change at the upstream and downstream junctions.

- **ICE Synthego analysis**

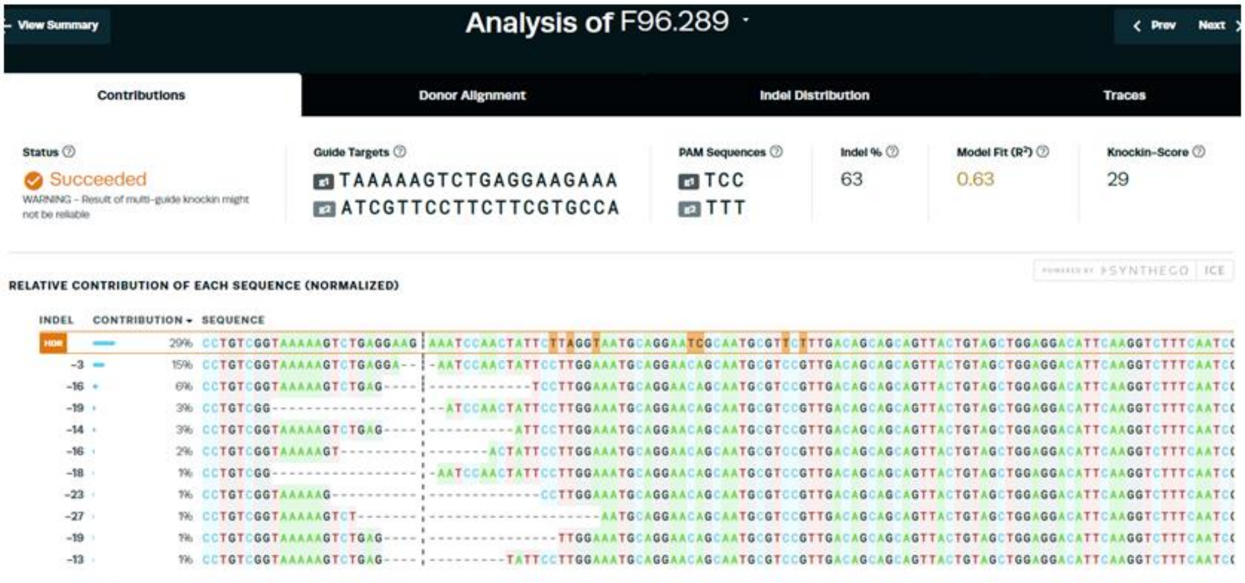

- **Miniseq analysis**

Plant	Total reads	GT efficiency (%)	Indel rate (%)
F96.289	74534	44.134	51.483

Figure S8. Identification and validation of *SlEPSPS1* GT0 event, F96.289. Top panel is a ICE-Synthego analysis that identified F96.289 as a potential GT event and subsequent confirmation by targeted NGS in the bottom panel

A

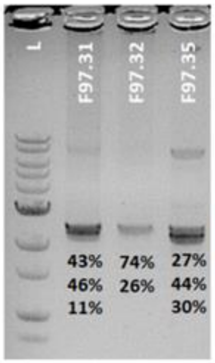

Calculation of normalized editing efficiency

No.	Event	Total miniseq reads	GT efficiency by miniseq (%)	Indel mutation efficiency by miniseq (%)	GT efficiency by Sanger seq, decomposed by ICE Synthego (%)	Approx. GT efficiency normalized to PCR bands (%)	Approx. indel mutation efficiency normalized to PCR bands (%)
1	F97.31	28969	39.622	59.560	11	17.1	74.0
2	F97.32	34513	28.059	71.892	6	20.6	79.2
3	F97.35	30264	99.134	0.819	16	26.4	73.2

B

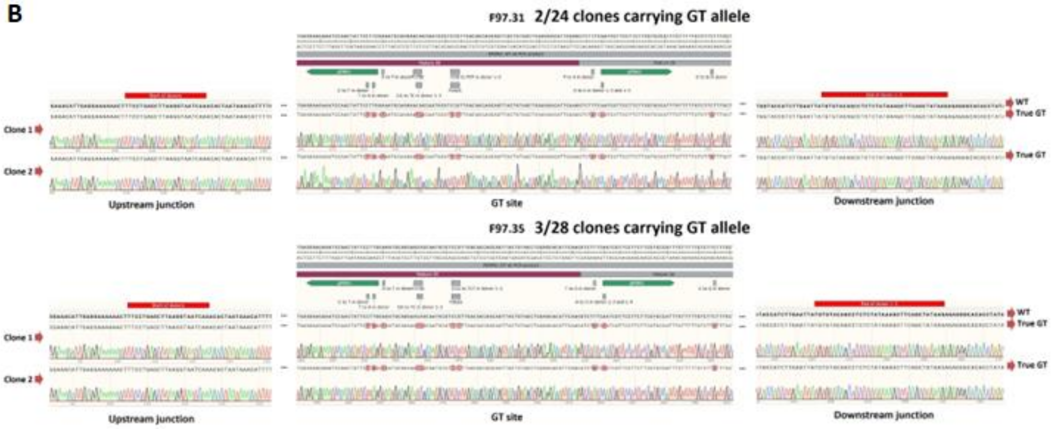

Figure S9. Identification and validation of the EPSPS1 GT events, F97.31, F97.32 and F97.35, obtained by the NKUDN-GT construct. **A** Calculation of normalized GT frequency. The targeted NGS results and resolved bands on agarose gel were used to identify the true GT frequency. Only the upper-most bands corresponding to WT-sized bands were considered to contain true GT alleles. The PCR bands on the agarose gel were measured by ImageJ and normalized to the band size to calculate the band frequency. **B** The potential EPSPS1 GT events, F97.31 and F97.35, were confirmed by pJET1.2 cloning and sequencing that show true GT allele carried by 2/24 clones (F97.31) and 3/28 clones (F97.35). Sequencing data revealed that 3/7 colonies contained desired base changes at the GT site and no additional change at the upstream and downstream junctions.

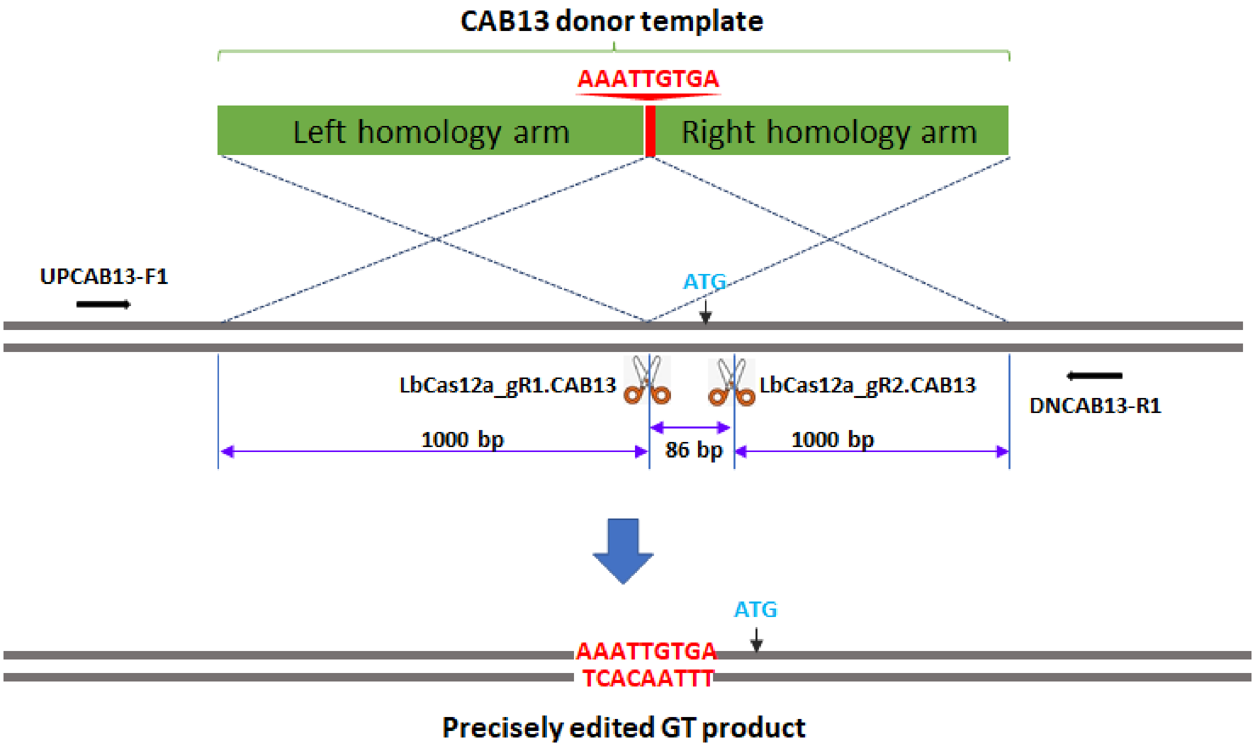

Figure S10. The donor and mode of allele replacement of the *SlCAB13*. Schematic diagrams describing the expected GT processes for exchanging the homologous DNA donor template with the genomic sequence at the *SlCAB13* locus. During the cloning of the *SlCAB13* donor, the AAATTGTGA sequence was introduced for integration into the genomic site. Two designated cutting sites were strategically planned to induce double-strand breaks at the targeted sites. The reverse and forward primers, represented by black arrows, are designed to amplify the targeted sites using a polymerase chain reaction. The diagrams were drawn not to their actual scales.

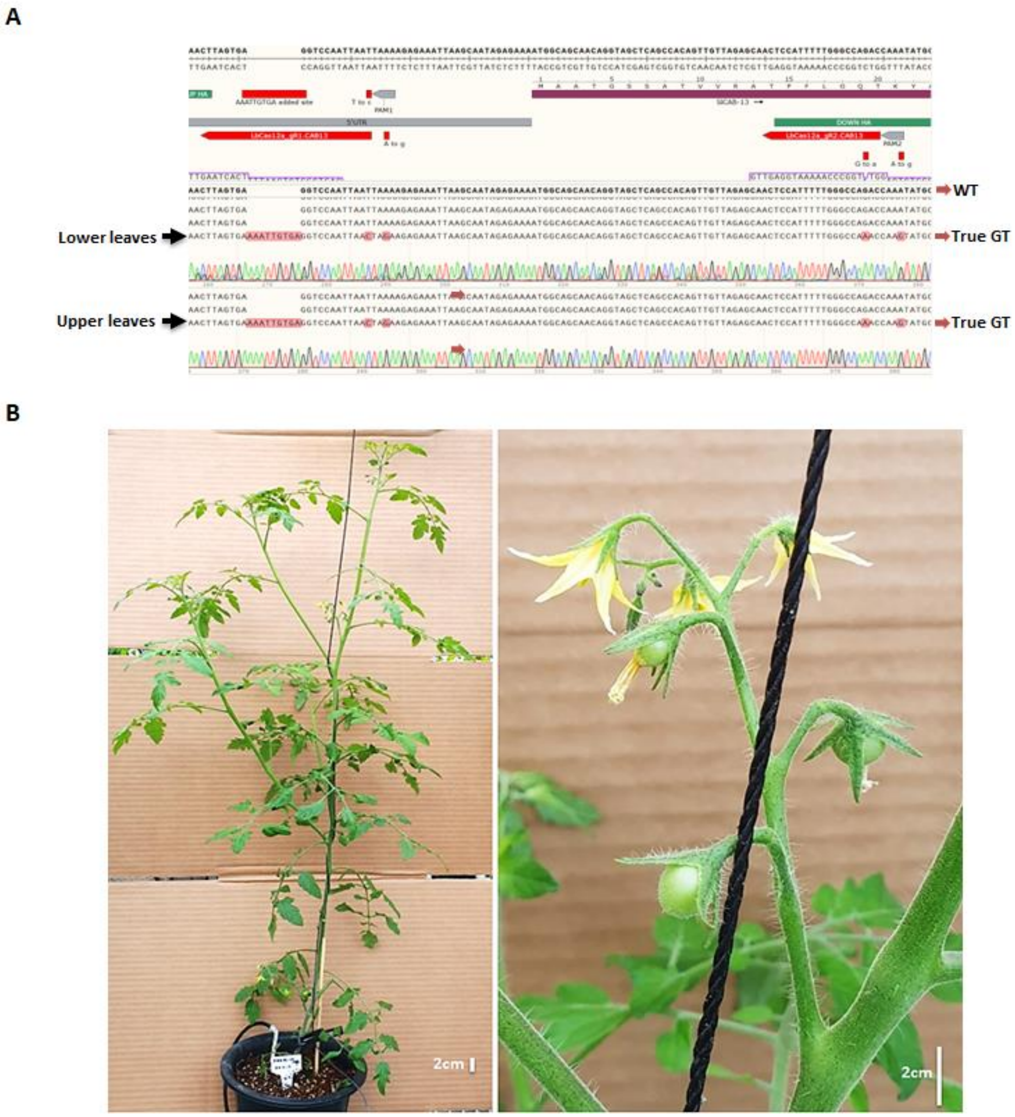

Figure S11. Design and evaluation of KUDN-based GT tools for *SlCAB13* editing. A Sequencing data revealed that 3/21 (~14.2%) colonies contained desired base changes at the GT site and no additional change at the upstream and downstream junctions. **B** The *SlCAB13* GT0 event planting in the greenhouse showed normal morphology during vegetative and reproductive stages.

A

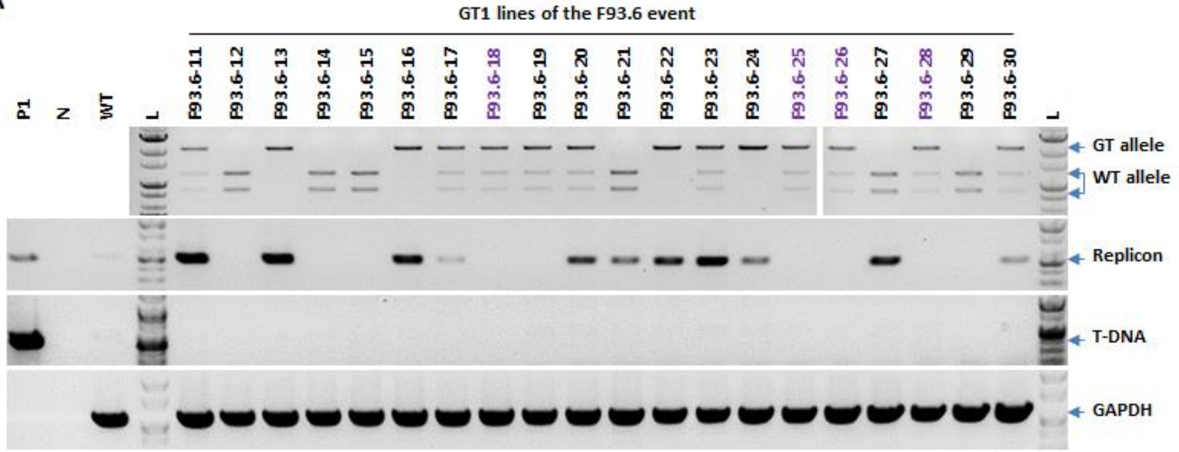
GT1 lines of the F93.6 event
P1
N
WT
L
F93.6-11
F93.6-12
F93.6-13
F93.6-14
F93.6-15
F93.6-16
F93.6-17
F93.6-18
F93.6-19
F93.6-20
F93.6-21
F93.6-22
F93.6-23
F93.6-24
F93.6-25
F93.6-26
F93.6-27
F93.6-28
F93.6-29
F93.6-30
L
GT allele
WT allele
Replicon
T-DNA
GAPDH

B

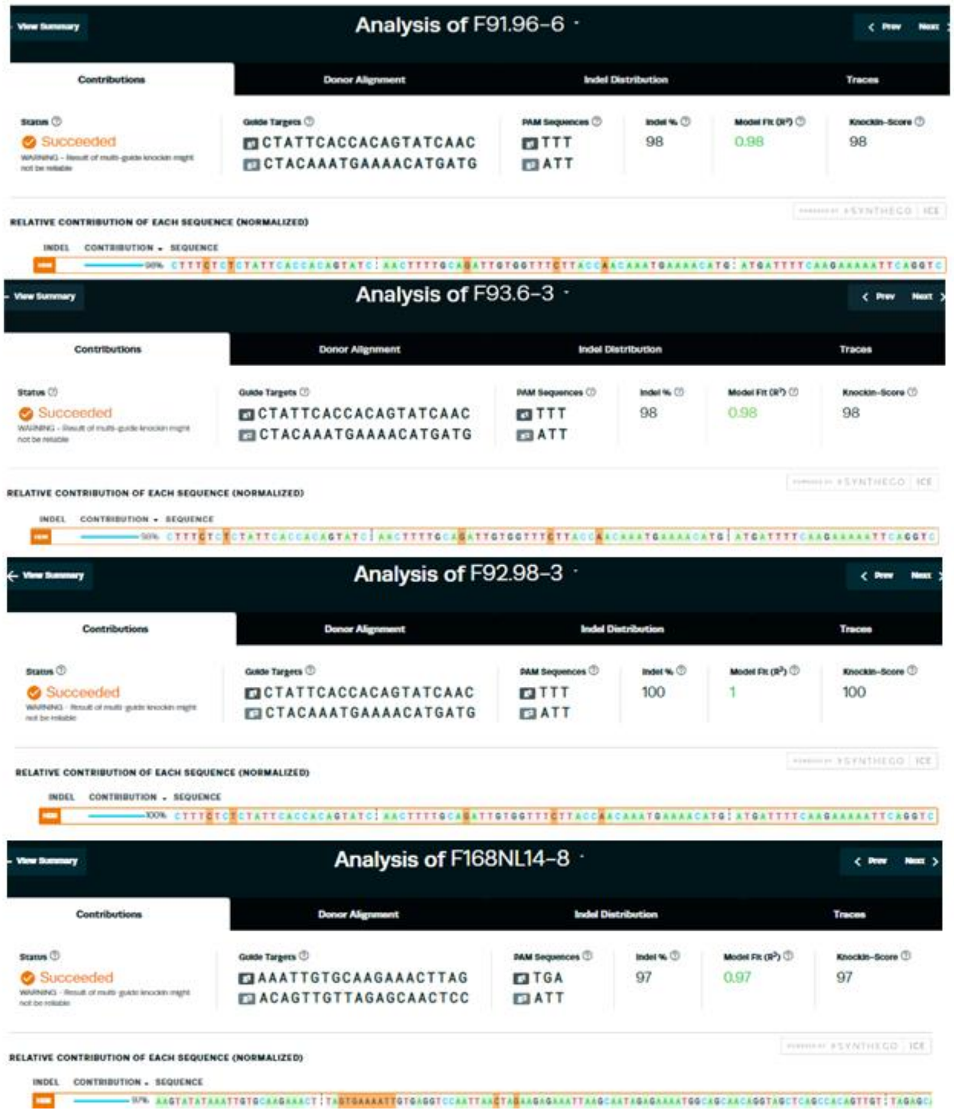
Analysis of F91.96-6
Contributions
Donor Alignment
Indel Distribution
Traces
Succeeded
CTATTCACCACAGTATCAAC
CTACAAATGAAAACATGATG
TTT
ATT
98
0.98
98
RELATIVE CONTRIBUTION OF EACH SEQUENCE (NORMALIZED)
Analysis of F93.6-3
Contributions
Donor Alignment
Indel Distribution
Traces
Succeeded
CTATTCACCACAGTATCAAC
CTACAAATGAAAACATGATG
TTT
ATT
98
0.98
98
RELATIVE CONTRIBUTION OF EACH SEQUENCE (NORMALIZED)
Analysis of F92.98-3
Contributions
Donor Alignment
Indel Distribution
Traces
Succeeded
CTATTCACCACAGTATCAAC
CTACAAATGAAAACATGATG
TTT
ATT
100
1
100
RELATIVE CONTRIBUTION OF EACH SEQUENCE (NORMALIZED)
Analysis of F168NL14-8
Contributions
Donor Alignment
Indel Distribution
Traces
Succeeded
AAATTGTGCAAGAAACTTAG
ACAGTTGTTAGAGCAACTCC
TGA
ATT
97
0.97
97
RELATIVE CONTRIBUTION OF EACH SEQUENCE (NORMALIZED)

Figure S12. The inheritance and segregation of GT alleles. **A** The inheritance of HKT1;2 GT allele in GT1 generation. CAPS assay was used to analyze the GT1 samples. **B** Representative plants carry homozygous GT alleles, which were revealed by sequencing and ICE Synthego analysis.

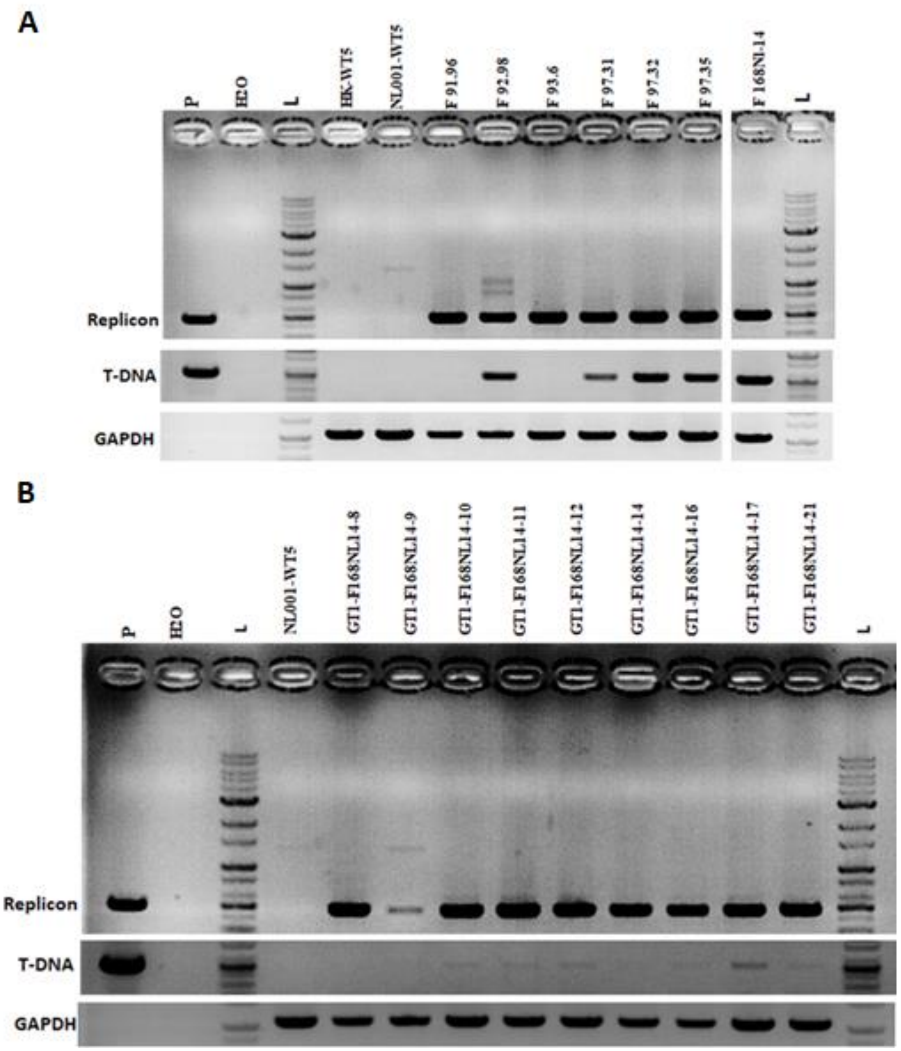

Figure S13. PCRs assessed T-DNA and replicon in the GT plants. A Representative GT0 events obtained in the study. **B** GT1 plants of the *SlCAB13* GT event F168NL14. The plasmids isolated from agro clones that carried the GT tool used for generating the events are referred to as positive control (P). H20 denotes the water control, while HK-WT5 and NL001-WT5 represent Hongkwang wild-type and NL001 wild-type, respectively. *SlHKT1;2* GT0 events were obtained using the GT construct without KUDN, with NKUDN, and with CKUDN, respectively, in Hongkwang's background and are referred to as F91.96, F92.98, and F93.6. Similarly, *SlEPSPS1* GT0 events were obtained using the GT construct with NKUDN in Hongkwang's background and are referred to as F97.31, F97.32, and F97.35. The *SlCAB13* GT0 event obtained using the GT construct with CKUDN in the NL001 background and referred to as F168NL14. GT1-F168NL14-8, 9, 10, 11, 12, 14, 16, 17, and 21 are GT1 plants carrying the homozygous *SlCAB13* GT allele.

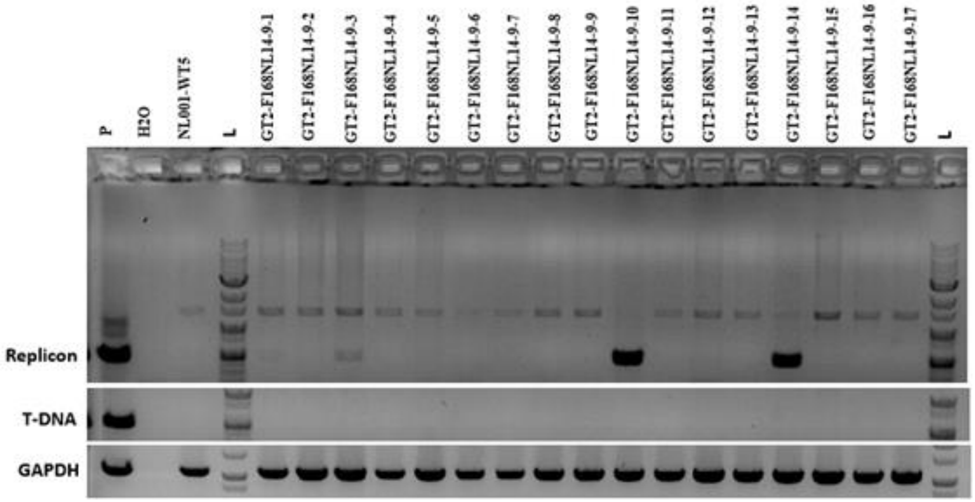

Figure S14. PCR-based assessment of T-DNA and replicon in the CAB13 GT2 plants. GT2 homozygous plants of the CAB13 GT1 line, F168NL14-9. The positive control (P) was GT plasmid isolated from Agro; water control was labeled H2O; and NL001 wild-type plant was marked as NL001-WT5. The GT2 homozygous plants from the F168NL14-9 GT1 line were marked as GT2-F168NL14-9-1, 2, 3, 4, 5, 6, 7, 8, 9, 10, 11, 12, 13, 14, 15, 16, and 17.

A

Locus	gRNA	Target	Chromosome	Position	Direction	Mismatches
EPSPS1	1	crRNA: TTTNCAAGGAATAGTTGGATTTCTTCC<br>DNA: TTTCCAAGGAAaAGTTGaATcTCTTCC	SL4.0ch05	60582371	-	3
CAB13	1	crRNA: TTTNATTAATTGGACCTCACTAAG<br>DNA: TTTAATTAATTGGcCCTCACTAca	SL4.0ch01	56418252	+	3
	2	crRNA: TTTNGTCTGGCCCAAAAATGGAGT<br>DNA: TTTGGTtTGGCCCAAAAATGGtGT	SL4.0ch12	4306011	-	2

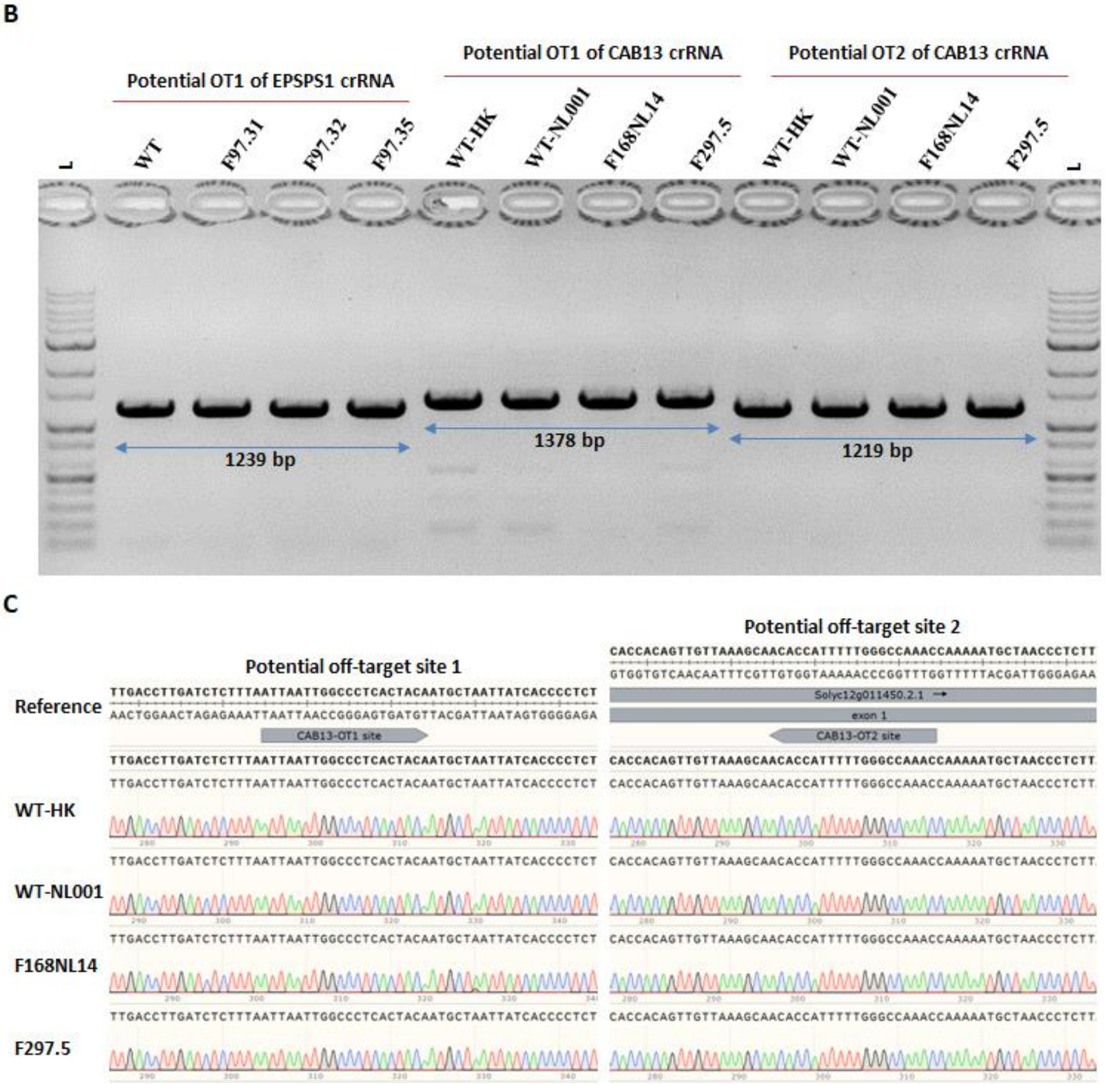

Figure S15. Assessment of potential off-target activities of the KUDN-based GT tools in the GT events. A Potential off-target sites of the gRNAs identified by Cas-offinder. **B** PCR products amplified from the selected potential off-target sites of *SlEPSPS1* and *SlCAB13* gRNAs in the GT events F97.31, F97.32, F97.35, and F168NL14 and other potential GT events (*SlEPSPS1*) and F297.5 (*SlCAB13*). **C** Chromatograms of the sequenced data show no editing traces at the potential off-target sites of *SlCAB13* gRNAs.
